# The costs, health and economic impact of air pollution control strategies: a systematic review

**DOI:** 10.1186/s41256-024-00373-y

**Published:** 2024-08-21

**Authors:** Siyuan Wang, Rong Song, Zhiwei Xu, Mingsheng Chen, Gian Luca Di Tanna, Laura Downey, Stephen Jan, Lei Si

**Affiliations:** 1 The George Institute for Global Health, Faculty of Medicine and Health, University of New South Wales, Sydney, NSW Australia; 2 Department of Epidemiology and Health Statistics, School of Public Health, Anhui Medical University, Hefei, China; 3 School of Medicine and Dentistry, Griffith University, Gold Coast, QLD Australia; 4 School of Health Policy and Management, Nanjing Medical University, Nanjing, China; 5 Jiangsu Health Vocational College, Nanjing, China; 6 Department of Business Economics, Health and Social Care, University of Applied Sciences and Arts of Southern Switzerland, Lugano, Switzerland; 7 School of Health Sciences, Western Sydney University, Campbelltown, NSW Australia; 8 Translational Health Research Institute, Western Sydney University, Penrith, NSW Australia

**Keywords:** Air pollution control, Cost–benefit analyses, Health co-benefits, Economic evaluation

## Abstract

**Background:**

Air pollution poses a significant threat to global public health. While broad mitigation policies exist, an understanding of the economic consequences, both in terms of health benefits and mitigation costs, remains lacking. This study systematically reviewed the existing economic implications of air pollution control strategies worldwide.

**Methods:**

A predefined search strategy, without limitations on region or study design, was employed to search the PubMed, Scopus, Cochrane Library, Embase, Web of Science, and CEA registry databases for studies from their inception to November 2023 using keywords such as “cost–benefit analyses”, “air pollution”, and “particulate matter”. Focus was placed on studies that specifically considered the health benefits of air pollution control strategies. The evidence was summarized by pollution control strategy and reported using principle economic evaluation measurements such as net benefits and benefit–cost ratios.

**Results:**

The search yielded 104 studies that met the inclusion criteria. A total of 75, 21, and 8 studies assessed the costs and benefits of outdoor, indoor, and mixed control strategies, respectively, of which 54, 15, and 3 reported that the benefits of the control strategy exceeded the mitigation costs. Source reduction (n = 42) and end-of-pipe treatments (n = 15) were the most commonly employed pollution control methodologies. The association between particulate matter (PM) and mortality was the most widely assessed exposure-effect relationship and had the largest health gains (n = 42). A total of 32 studies employed a broader benefits framework, examining the impacts of air pollution control strategies on the environment, ecology, and society. Of these, 31 studies reported partially or entirely positive economic evidence. However, despite overwhelming evidence in support of these strategies, the studies also highlighted some policy flaws concerning equity, optimization, and uncertainty characterization.

**Conclusions:**

Nearly 70% of the reviewed studies reported that the economic benefits of implementing air pollution control strategies outweighed the relative costs. This was primarily due to the improved mortality and morbidity rates associated with lowering PM levels. In addition to health benefits, air pollution control strategies were also associated with other environmental and social benefits, strengthening the economic case for implementation. However, future air pollution control strategy designs will need to address some of the existing policy limitations.

**Supplementary Information:**

The online version contains supplementary material available at 10.1186/s41256-024-00373-y.

## Background

Air pollution is a major environmental and public health problem affecting millions of people worldwide [1]. According to the World Health Organisation (WHO), it is among the leading causes of mortality, with exposure to indoor and outdoor air pollution associated with approximately 6.7 million premature deaths in 2019 [2]. In addition to its health impacts, air pollution has environmental, ecological, and economic consequences [3]. For example, one economic impact relates to the substantial costs associated with treating and managing air pollution-induced illnesses [4, 5], as well as indirect societal expenditures resulting from the loss of productivity due to reduced working days [6]. The World Bank estimated that the overall cost of air pollution on health and well-being was approximately $8.1 trillion U.S. dollars, or 6.1% of GDP, in 2019 [7].

The need to reduce the environmental and health impacts of air pollution has been recognized for several decades. Many developed countries have implemented comprehensive multi-pollutant control strategies aimed at mitigating the health effects of key pollutants, including particulate matter (PM), ozone, nitrogen dioxide, and sulfur dioxide [8, 9]. In recent years, developing countries with large populations have also begun tightening air quality standards. For example, China implemented the National Clean Air Action Plan (2013–2017) and followed it with the Three-Year Action Plan for Clean Air starting in 2018 to jointly lower emissions from various pollution sources [10, 11]. Health assessment studies have consistently highlighted the substantial health and economic benefits associated with reducing air pollution through these measures [12–14].

Despite the substantial health benefits of air quality control strategies, their implementation comes at a cost. The magnitude of benefits and costs is primarily dependent on the relative nature of the control strategy, the size and setting of the intervention, the specific exposure and health endpoints considered, and the assumptions of the underlying economic evaluation [15]. Some high-income countries require a regular assessment of the relative costs and benefits of proposed environmental regulations, including air pollution regulations. For example, the US Environmental Protection Agency (EPA) has been required by law to conduct several comprehensive cost–benefit analyses of the Clean Air Act [16].

On a global scale, there is a gap in the systematic analysis of the costs and health benefits of air pollution control strategies. While the evidence base strongly supports that lowering exposure to air pollution is beneficial to health and reduces the burden on health systems, air pollution control strategies often come at significant costs. Thus, there is an imperative need to understand the relative costs and benefits of such interventions to ensure evidence-based air policies, particularly in resource limited settings. This study sought to fill this gap by systematically reviewing the economic impact of air pollution control strategies. The objective was to identify successful pollution control strategies, summarize economic evaluation methodologies, and highlight existing policy limitations. The findings are intended to inform the design of more optimal and targeted air policies, particularly in low- and middle-income country (LMIC) settings where there is a critical need to deliver cost-effective interventions to control pollution.

## Methods

### Search strategy

Six databases, including PubMed, Scopus, The Cochrane Library, Embase, Web of Science, and the CEA registry, were searched using a predefined strategy developed by combining keywords such as “air pollution”, “particle matter”, and “cost–benefit analyses”. The searches included the period from each database's inception to November 2023, without limitations on study design or region. Detailed summaries of the strategy search strategies are shown in Online Appendix 1.

### Study selection, eligibility, and exclusion criteria

The database searches identified studies that explored the public health impact of air pollution control strategies, focusing on those that specifically assessed health benefits as part of the cost–benefit evaluation. Studies were included in the analysis if they: 1) were economic evaluation studies (cost–benefit analysis) of air pollution control strategies; 2) reported health and economic benefits of air pollution control strategies; and 3) were published in English. Studies that were not peer-reviewed articles, such as government reports or conference abstracts, were excluded.

### Data extraction

Two reviewers (SW and RS) independently screened the title, abstract, and full text of each study. Conflicts were resolved through consultations with a third reviewer (LS). Information from the final included studies was gathered using a data extraction sheet developed following the initial phase of the literature review. The following data elements were extracted: study identification information (authors, year of publication, and country of conduct), study design (perspective, scope, and settings), type of intervention (outdoor intervention, indoor intervention, or mixed intervention), pollution control method (source reduction methods or end-of-pipe treatments), pollution control strategy category, pollutant type targeted, study methodologies (methodologies that modeled emissions, estimated costs, and estimated benefits), cost estimates, benefit estimates, cost–benefit estimates and sensitivity analysis estimates. A full list of the extracted elements is provided in Online Appendix 2.

### Synthesis

A narrative synthesis was used to summarize the findings. Economic evidence were summarized using standard cost–benefit measurements that define an intervention as effective if the net benefit (total benefit minus total cost) is positive or the benefit–cost ratio (total benefit divided by total cost) is > 1 [17]. We followed the general principles for evidence synthesis reviews and reported the findings using PRISMA reporting guidelines (Online Appendix 3) [18].

### Quality appraisal and risk of bias assessment

The Consolidated Health Economic Evaluation Reporting Standards 2022 (CHEERS 2022) reporting guidance for economic evaluations was used to conduct a risk of bias assessment [19]. CHEERS 2022 includes 28 items, all of which were used to assess the quality of the included studies. We assessed the quality of evidence following the reporting guidance from the CHEERS 2022 Explanation and Elaboration report [20]. In the absence of a validated scoring system for the checklist, a qualitative assessment of the completeness of reporting for each item was conducted [19].

## Results

### Characteristics of the included studies

**Fig. 1:**
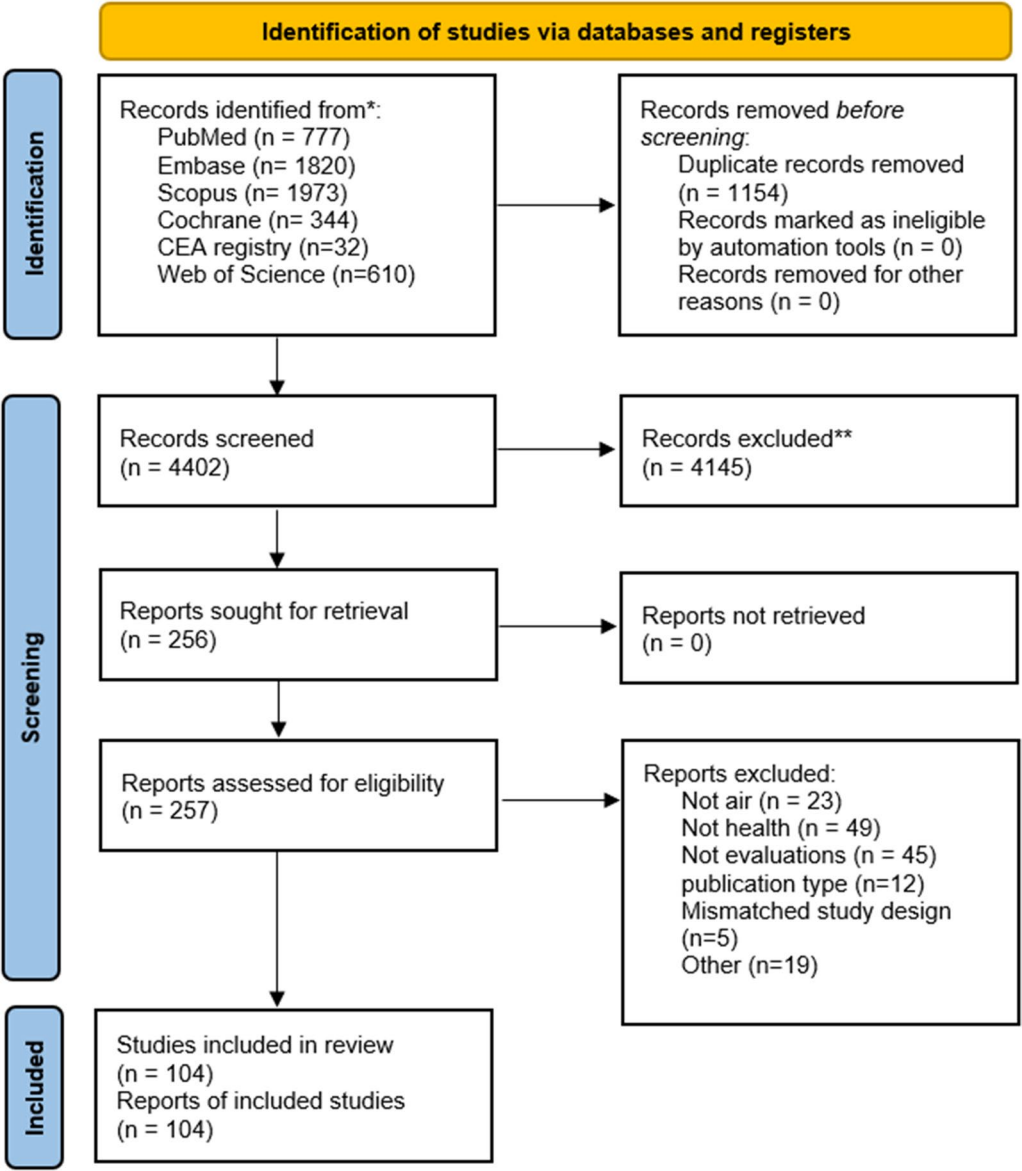
PRISMA flow diagram of study selection

The search strategy yielded 4966 records across the six databases, from which 4,402 unique records were identified for title, abstract, and full-text screening. A total of 104 studies were ultimately found to meet the inclusion criteria. The selection process, developed using the PRISMA flowchart, is shown in Fig. 1.

Economic evaluation studies were identified that examined the cost–benefit ratio of several air pollution control strategies across various countries, with some dating back over 50 years. Overall, there was a relatively balanced distribution of studies conducted in low- and middle-income settings as well as high-income settings (n = 48 and 47, respectively), and most studies were published within the last decade (n = 74). Outdoor interventions, which sought to reduce local or ambient air pollution, were the most common type of pollution control strategy (n = 75; 72%). Meanwhile, 21 studies assessed the cost–benefit ratio of indoor interventions that aimed to lower exposure at the individual or household level. A total of eight studies evaluated control strategies that incorporated both indoor and outdoor interventions. Most pollution control strategies sought to mitigate emissions or pollutants directly from their origin (n = 42), while others employed end-of-pipe treatments to reduce pollution after its release, often through the use of filtration systems, scrubbers, or other pollution control devices (n = 15). A table of the included studies is shown in Online Appendix 4 and the study characteristics are summarized in Table 1.

**Table 1:** Summary characteristics of included studies

Country setting	Total	Positive cost–benefit	Partial positive cost–benefit***	Negative cost–benefit
HICs	47	34	7	6
LMICs	48	29	13	6
Mixed regions	9	9	0	0
*Year of publication*				
From inception to 2012	30	26	3	1
2013–2022	74	46	18	10
*Typology of air control strategies*				
Outdoor interventions	75	54	13	8
Indoor interventions	21	15	4	2
Mixed interventions	8	3	4	1
*Pollution control method*				
Source reduction	42	26	10	6
End-of-pipe treatment	15	11	4	0
Mixed methods	17	9	6	2
Not specified	30	26	1	3
*Pollutant strategies*				
Single pollutant strategy	51	39	8	4
Multi-pollutant strategy	48	32	11	5
Not specified	5	4	1	0
Pollutants targeted*				
PM (PM2.5, PM10 and other forms)	84	61	16	7
O_3_	19	16	2	1
NO_X_	34	19	9	6
SO_X_	32	17	12	3
Health related endpoints*				
Premature deaths	53	40	8	5
Restricted activity days	18	14	2	2
Cardiovascular disease	43	31	6	6
Asthma	14	10	1	3
COPD	22	16	3	3
Lung cancer	21	13	4	4
Other respiratory diseases**	44	34	7	3
Other health endpoints and not specified	13	9	2	2

^*^Studies can concurrently assess multiple pollutants and health endpoints

^**^Excluding Lung cancer, asthma, and COPD (Chronic Obstructive Pulmonary Disease)

^***^We classified studies as having reported partially positive cost–benefit results if they analyzed multiple interventions and presented a mix of positive and negative cost–benefit outcomes among these interventions

### Pollution control strategies by category

Pollution control strategies involving a variety of control methods aimed at reducing both outdoor and indoor pollution were identified. Specific examples of outdoor interventions included transitions to cleaner energy and fuel sources [21, 22], tighter vehicle emission regulations [23], and improved agriculture practices and technologies such as intercropping and low-emissions animal housing systems [24, 25]. Another type of outdoor pollution control method was the use of end-of-pipe treatments for high-emission sources, such as retrofitting coal-fired power plants with scrubbers [26] or using particle filters and oxidation catalysts for diesel vehicles [27]. Common indoor pollution control strategies included interventions that encourage the use of cleaner and improved stoves [28, 29], and promoting clean air ventilators in workplaces and households [30]. Air pollution control strategies grouped by intervention type and pollution control methodology are summarized in Table 2.

**Table 2:** Summary of air pollution control strategies by intervention type and pollution control methodology

	Pollution control method	
	Source reduction or prevention measures	End-of-pipe treatments
Outdoor interventions	Forestry and agricultural measures [24, 25, 31–33]: manure application technology, low emissions animal housing, intercropping, smarter livestock feeding strategies, fertilizer substitution	Vehicle emission reduction technology [27, 34–37]: Retrofitting diesel vehicle filters/diesel oxidation catalysts Point emission reduction [38–41]: carbon filtration for ozone removal, electrostatic precipitators on stationary sources, retrofitting power plants with dieselization (scrubbers)/denitrification technology, selective catalytic reduction and dust removal technology
	Road, off-road and sea transport [23, 42–53]: Low emission zones, road pricing, alternative fuels (vehicle and shipping), Inspection & Maintenance programs, vehicle retirement programs, Electric vehicle subsidy/mandate	
	Global Climate Change policies [54–63]: Paris 2 °C agreement	
	Emission and energy standards/caps [21, 26, 64–83]: Cap on coal consumption, emission ceilings, cap-and-trade policy, polluter pays principle, coal-fired power plant closures	
	Cleaner/Alternative energy source [22, 84–88]: Renewable energy, power plant efficiency abatements	
Indoor interventions	Household clean heating [89–93]	Indoor air quality control technology [30, 94–98]: air cleaners, indoor air particle filters, air ventilators
	Household cooking strategies [28, 29, 99–106]: Liquefied petroleum gas, natural gas, biogas, electric stoves, improved cooking stoves	
Mixed interventions	Multicategory control strategies: Cap on Coal Consumption, transport regulations, cleaner energy, improved stoves, clean heating [107–113]	

### Economic evaluation modeling of air pollution control strategies

**Fig. 2:**
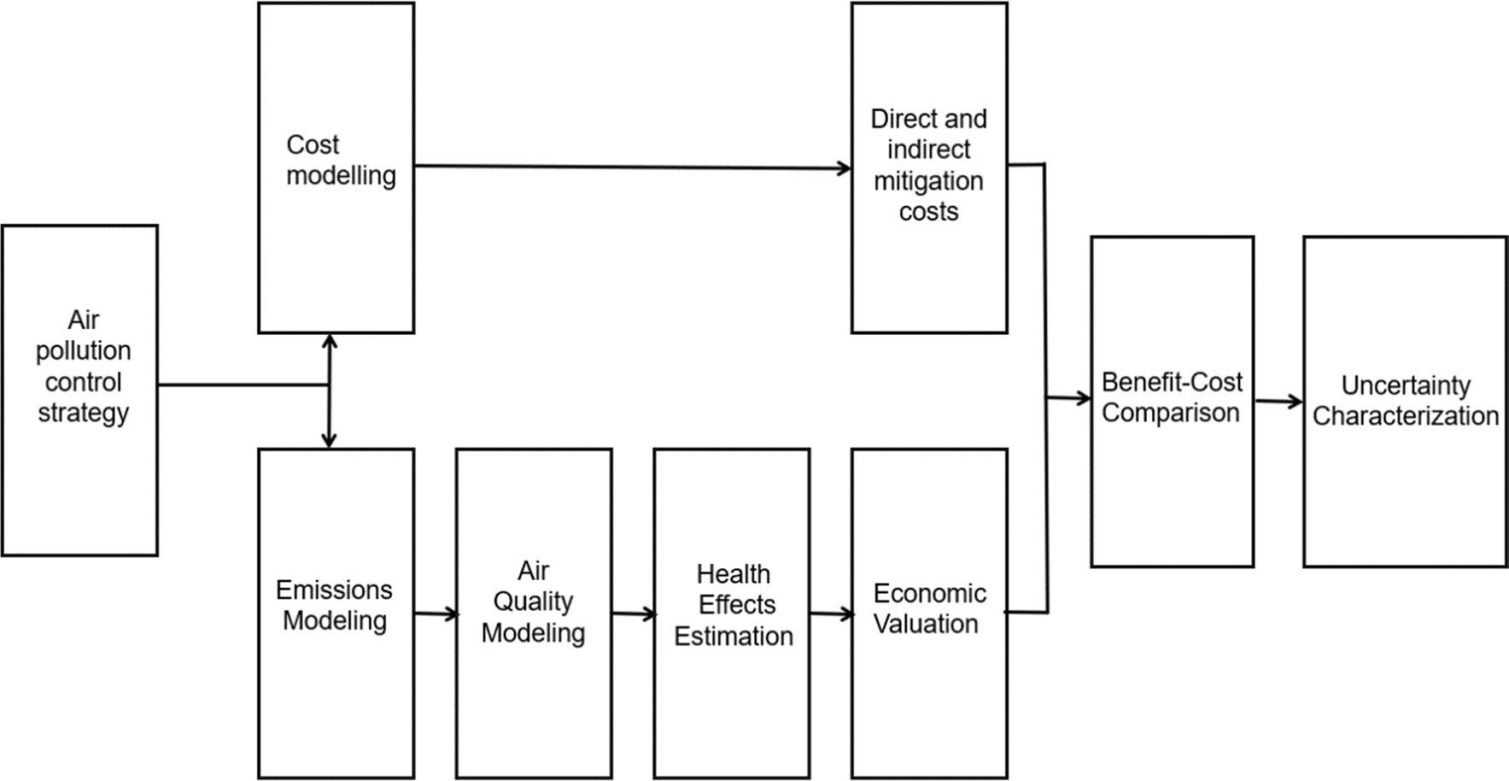
Analytical sequence for the economic evaluation of air pollution control strategies

The Impact Pathway Approach (IPA) [114], which connects interrelated modules for different aspects of the evaluation process, was commonly used to evaluate the effects of ambient air pollution on human health. This is a multistep approach that establishes links between emissions, exposure, and effects by estimating pollutant emissions and dispersion, then modeling exposure of the target population to assess health impacts, quantify the costs, and compare the benefits and mitigation costs. While methodologies for estimating costs and benefits varied by intervention and study context, most studies employed dose–response parameters to assess health gains from reduced pollution exposure. Subsequently, economic evaluation modeling techniques, such as the Value of Statistical Life (VSL) or Cost of Illness (COI), were employed to quantify the economic health benefits. A summary of the evaluation process, including the emissions, chemical transport, and health assessment models, as well as the cost–benefit assessment, are shown in Fig. 2.

The IPA also uses Integrated Assessment Models (IAMs) to assess the health impacts of a broad range of policy scenarios or technological interventions. IAMs incorporate geographical, populational, and industry-specific data to estimate the emission and dispersion of primary and secondary pollutants and model populational exposure to assess health and economic impacts. The choice of modules was largely dependent on the specific setting of the study, as well as the control policy being considered. For example, the Global Change Assessment Model (GCAM) and the Greenhouse Gas and Air Pollution Interactions and Synergies (GAINS) model were two commonly used IAMs for estimating the impact of both air pollution and climate change-related policies on emissions. In addition, the Comprehensive Air Quality Model with Extension (CAMx) and the Community Multiscale Air Quality (CMAQ) model were often used to model pollutant atmospheric concentrations, while the Benefits Mapping and Analysis Program (BENMAP) was used to assess health impacts.

Costs associated with air pollution interventions encompass several elements. These include initial investment costs, such as research and development of cleaner technologies [84], as well as operating and maintenance expenses, such as heavy vehicle inspection and maintenance programs [43]. Finally, mitigation costs are compared against intervention benefits using standard economic evaluation metrics such as computing net benefits or benefit–cost ratios.

### Health benefit assessment

Most studies used dose–response parameters to predict health outcomes from changes in exposure and then compared the money saved by health gains to the costs of mitigation. However, the choice of parameters varied depending on the nature of the exposure, the setting of the study, and the selected health endpoints. Most of the studies focused on evaluating the economic benefit of lowering particulate matter (n = 84), which is considered the most important factor affecting human health. Other hazardous gases, including NO_X_, SO_X_, and O_3_ (n = 34, 32, 19, respectively), were also considered. Premature deaths, cardiovascular diseases, and respiratory diseases (chronic obstructive pulmonary disease, lung cancer, chronic bronchitis, and ischaemic heart disease) were the most widely assessed health endpoints (n = 53, 43, and 44, respectively). Some studies also considered the benefit of increased productivity from a drop in the number of restricted working days (n = 18). In studies evaluating the economic health benefit of reducing premature deaths, the VSL approach was the most common methodology used. The Willingness to Pay (WTP) and COI methods were also used to quantify disease burden, and the Human Capital (HC) approach was used to evaluate losses in productivity.

### Economic impact of air pollution control strategies

There was widespread economic evidence in support of implementing air pollution controls. Table 3 summarizes the cost-benefit results by pollution control category. Of the 104 studies analyzed, 72 (69%) reported that the benefits of the control strategy outweighed the costs. Most studies evaluated outdoor interventions, with 54 of 75 finding positive evidence in favor of these interventions. Of the 21 studies assessing indoor interventions, 15 showed positive results. Eight studies examined the cost–benefit ratio of both outdoor and indoor interventions, of which three reported net positive results. The number of studies that reported benefits exceeding costs, benefits exceeding costs for parts of the intervention, and costs exceeding benefits are presented in Table 1. Except for transport regulations, the pollution control categories showed consistently positive economic results. Of the 13 studies assessing transport regulations, only three reported positive outcomes, while six indicated mixed results and four reported negative cost–benefit outcomes. In 41 studies investigating the impact of uncertainties on cost–benefit outcomes, several key variables were consistently analyzed, including discount rates, VSL figures, cost parameters, and dose–response models. In some instances, adopting lower VSL figures and projecting higher mitigation costs helped to shift the economic assessment of the intervention from cost-beneficial to non-cost-beneficial [22, 48, 58, 115, 116].

**Table 3:** Summary of cost–benefit results by pollution control category

Outdoor interventions	Total	Positive cost–benefit	Partial positive cost–benefit*	Negative cost–benefit
Forestry and agricultural measures	5	4	1	0
Road, off-road and sea transport	13	3	6	4
Global climate change co-benefits	10	9	0	1
Emission standards/caps	22	17	2	3
Cleaner alternative energy	6	6	0	0
Vehicle emission reduction technology	5	4	1	0
Point emission reduction	4	3	1	0
Multi method outdoor interventions	7	5	2	0
Not specified	4	4	0	0
*Indoor interventions*				
Household clean heating	5	4	0	1
Household cooking strategies	10	7	2	1
Indoor air quality control technology	6	4	2	0
Mixed intervention types				
Multicategory	7	2	4	1
Total	103	71	21	11

^*^We classified studies as having reported partially positive cost–benefit results if they analyzed multiple interventions and presented a mix of positive and negative cost–benefit outcomes among these interventions

### Social, environmental, and ecological benefits

A total of 32 studies [14, 25, 29, 31–33, 42, 47, 48, 52, 60, 61, 73, 84, 87, 92, 99–108, 116–121] considered the broader social, environmental, or ecological benefits of pollution control strategies. Of these, 16 studies [25, 29, 33, 42, 47, 48, 52, 84, 100, 102–107, 120] estimated the environmental benefits of reducing CO_2_ emissions by employing a carbon market price or CO_2_ abatement cost. Other studies (n = 18) valued the additional morbidity improvements and productivity gains from reducing the number of restricted days and increasing the number of working days. Krewitt et al. [117] used exposure–response functions from open-top chamber experiments to quantify the economic benefit of increased crop yield from reduced SO_2_ emission. Partially positive or positive cost–benefit results were demonstrated in 31 of the 32 studies. In addition, nine out of 10 studies showed that environmental policies, particularly long-term policies aimed at mitigating greenhouse gas emissions, may also have short-term secondary air pollution benefits, contributing to positive economic evidence in support of the policy.

### Risk of bias assessment and quality appraisal of evidence

The results of the quality assessment under the CHEERS 2022 framework are shown in Online Appendix 5. All studies reported on items 6 and 7, providing relevant contextual information regarding the setting, location, and intervention or scenario of consideration. Most studies (n = 60, 74, 85, 72, respectively) adhered to the reporting criteria for items 1, 2, 3, and 9 (title, abstract, background, and time horizon). Additionally, a total of 85, 100, 99 and 88 studies reported on the selection, measurement and valuation of outcomes and costs (items 11, 12, 13 and 14, respectively). Few studies (n = 6, 2) considered the heterogeneity and distributional effects of the outcomes (items 18 and 19). No studies reported on items 8, 21, and 25 (perspective, engagement with patient, and effects of engagement with patients). Meanwhile, a total of 44 and 41 studies characterized and reported on uncertainty (items 20 and 24), and a total of 59 and 42 studies disclosed the funding source and competing interests, respectively (items 27 and 28).

## Discussion

Our review of the economic evidence suggests that economic assessments of air pollution control strategies face several key uncertainties at each stage of the evaluation process, including emissions projection, exposure modeling, and quantification of the benefits and costs. Cost uncertainties primarily stemmed from the cost data, the cost model, and the choice of discounting factors for operating and maintenance costs. The uncertainties relating to benefit estimation were considerably larger. Two commonly acknowledged factors across all studies were the choice of an appropriate Concentration Response Function (CRF) to estimate the health effects of exposure and the selection of a VSL figure to monetize health gains. Differences in air pollutant composition, population age structure, and the quality of public health systems contributed to varying exposure-effect relationships across different populations and regions. Thus, it is critical to select concentration–response functions that are tailored to the specific context of each study. The choice of appropriate VSL and CRF proved particularly challenging for many studies conducted in low- and middle-income settings that lack supporting epidemiologic and economic evidence. Many of these studies used the benefits transfer method to estimate an approximate figure by adjusting VSL estimates from developed countries, despite existing literature showing the limitations of this approach [109]. Other studies used concentration–response functions established from epidemiologic studies in developed countries that may not reflect the appropriate populational or environmental context. The choice of valuation methods also greatly influences the benefits estimation. For example, studies employing contingent valuation estimates may inadvertently overstate the economic benefits, while those utilizing the COI approach may not fully encompass all economic benefits [122].

We find that studies measuring both economic and health benefits were more likely to report positive economic results from the control strategies. However, the methods varied in the types and sizes of social and environmental benefits considered. For example, the environmental benefits from reduced carbon dioxide emissions and time savings associated with indoor cooking interventions generally outweighed the corresponding health benefits. This was not typically the case for outdoor interventions. In some studies [101], the standalone health benefits were insufficient to cover mitigation costs, while the addition of social benefits resulted in net positive results. These findings highlight the importance of an integrated or holistic approach in the evaluation framework.

While this study highlighted overwhelming economic evidence in support of various air pollution control strategies, it also revealed a need to address policy limitations and barriers. This includes ensuring equality among different socioeconomic and geographical populations. Air pollution is a major cause of health inequalities worldwide, particularly for women, elders, and people of low socioeconomic status [123–125]. Thus, future control policies and policy evaluations will need to target these priority groups. Despite the epidemiologic evidence demonstrating the disproportionate health impacts of air pollution on elders and infants, only six of the 104 studies included in this review considered the distributional effects and heterogeneity of outcomes on different subpopulations [124, 126]. While air pollution has a similar impact on the health of men and women, particular occupational or social norms can lead to disproportionately high levels of exposure among some groups of women, such as housewives who are using inefficient stoves in low- and middle-income settings. This suggests a need for targeted interventions and evaluations in this population [28]. Despite overall net positive outcomes for society, specific cohorts, particularly rural populations, or people living in regions of low socioeconomic status, may experience net economic losses due to disproportionately high mitigation costs [93]. Clean air has substantial positive health and social benefits that spill over to society. However, without government subsidies, costs are disproportionately borne by individuals or private sectors, posing challenges to implementation [105]. Thus, economic evaluations should consider assessing the private and social cost-benefits separately.

This study had a few limitations. First, the review was limited to peer-reviewed articles, potentially omitting relevant grey literature. The lack of all available information, including government documents that evaluate environmental air interventions, may contribute to a biased or incomplete interpretation of the full economic evidence. Second, this study has potential publication bias, including funding biases from governments or organizations with vested interests and the selective reporting of studies with positive health and economic outcomes. These biases may skew the overall economic results in favor of certain policies and underrepresent alternative approaches or outcomes. Third, due to variability in outcome measurements and analytical methodologies used by the included studies, it was not feasible to conduct a meta-analysis or otherwise quantitatively synthesize the overall economic evidence.

## Conclusions

This study systematically reviewed economic evidence on the costs and benefits of air pollution control strategies across different countries and timeframes. Nearly 70% of the studies reported data in support of the control policies, with particularly strong economic evidence identified by those using a broader benefits framework. While there was broad economic support for air pollution control in general, the findings also underscore the scarcity of economic and epidemiological evidence needed to substantiate such economic evaluations, particularly within LMICs. In addition, there is a pressing need to prioritize environmental and economic equity in the development of targeted interventions, especially among vulnerable populations in LMICs who are at higher risk for air pollution-related illness due to existing geographical, health, or socioeconomic disparities. The insights gained from this review will help to inform the design of future air pollution control policies and the economic evaluations of related interventions.

### Supplementary Information

Additional file 1.

Additional file 2.

Additional file 3.

Additional file 4.

Additional file 5.

## Data Availability

The data used and/or analyzed during the current study are extracted from included studies and are available from the corresponding author on reasonable request.

## Abbreviations

EPA: Environmental Protection Agency
IPA: Impact Pathway Approach
IAMs: Integrated Assessment models
GCAM: Global Change Assessment Model
GAINS: Greenhouse Gas and Air Pollution Interactions and Synergies
CAMX: Comprehensive Air Quality Model with Extension
CMAQ: Community Multiscale Air Quality model
BENMAP: Benefits Mapping and Analysis Program
LAP: Local Air Pollution
GCC: Global Climate Change
VSL: Value of Statistical Life
CRF: Concentration Response Function
WTP: Willingness to pay
COI: Cost of Illness
HC: Human Capital
LMICs: Low- and middle-income countries
HIC: High income countries
PM: Particle matter
COPD: Chronic Obstructive Pulmonary Disease
