# Supplementary material for: The costs, health and economic impact of air pollution control strategies: a systematic review
Source: Glob Health Res Policy. 2024 Aug 21;9:30. doi: 10.1186/s41256-024-00373-y (PMC11337783; doi:10.1186/s41256-024-00373-y)
Supplement: Supplementary file 1 — Additional file 1. [file 41256_2024_373_MOESM1_ESM.docx]

**Pubmed**

|  | **Search terms** | **No. of hits** |
| --- | --- | --- |
| #1 | "air pollution/analysis"[MeSH Major Topic] OR "air pollution/economics"[MeSH Major Topic] | 17393 |
| #2 | "air pollution, indoor/analysis"[MeSH Terms] OR "air pollution, indoor/economics"[MeSH Terms] | 8659 |
| #3 | "traffic related pollution/analysis"[MeSH Terms] OR "traffic related pollution/economics"[MeSH Terms] | 62 |
| #4 | "air pollut*"[All Fields] OR "air quality"[All Fields] OR "urban pollut*"[All Fields] OR "ambient air pollution"[All Fields] OR "atmospheric pollut*"[All Fields] OR "air contamination"[All Fields] OR "ambient particulate matter"[All Fields] OR "air pollution control"[All Fields] OR "air-pollution"[All Fields] | 161919 |
| #5 | "nitrogen dioxide"[All Fields] OR "nitrates"[All Fields] OR "ozone"[All Fields] OR "O3"[All Fields] OR "NO2"[All Fields] OR "Sulfur Dioxide"[All Fields] OR "SO2"[All Fields] | 133213 |
| #6 | "particulate matter"[All Fields] OR "particulate matter"[MeSH Terms] OR "Airborne particulate matter"[All Fields] OR "Air Pollutants"[All Fields] OR "pm"[All Fields] OR "ultrafine particulate matter"[All Fields] OR "UFPM"[All Fields] | 243383 |
| #7 | "Vehicle Emissions"[MeSH Terms] OR "Vehicle Emissions"[All Fields] OR "low emission zone"[All Fields] | 11991 |
| #8 | #1 OR #2 OR #3 OR #4 OR #5 OR #6 OR #7 | 387909 |
| #9 | "Cost-Benefit Analysis"[MeSH Terms] | 90860 |
| #10 | "cost-effectiveness analysis" OR "cost benefit analysis" OR "cost minimization analysis" OR "cost-utility analysis" OR "rate of return" | 98857 |
| #11 | #9 OR #10 | 98857 |
| #12 | #8 AND #11 | 777 |

**Embase**

|  | **Search terms** | **No. of hits** |
| --- | --- | --- |
| #1 | air pollu*.mp. [mp=title, abstract, heading word, drug trade name, original title, device manufacturer, drug manufacturer, device trade name, keyword heading word, floating subheading word, candidate term word] | 135,687 |
| #2 | exp air pollutant/ | 98,248 |
| #3 | *air control/ or *air particle control | 2,916 |
| #4 | atmospheric dispersion/ | 5,478 |
| #5 | nitrous oxide emission/ | 1,429 |
| #6 | indoor air pollution/ | 14,786 |
| #7 | exp nitrogen oxide emission/ | 1,562 |
| #8 | ozone depletion/ | 941 |
| #9 | (“cost-benefit” or “cost effectiveness” or “cost utility” or “cost minimization” or “rate of return” or “net present value” or “NPV”).mp. [mp=title, abstract, heading word, drug trade name, original title, device manufacturer, drug manufacturer, device trade name, keyword heading word, floating subheading word, candidate term word] | 318,514 |
| #10 | 1 or 2 or 3 or 4 or 5 or 6 or 7 or 8 | 185,209 |
| #11 | economic evaluation/ or “cost benefit analysis”/ or “cost effectiveness analysis”/ or “cost minimization analysis”/ or “cost utility analysis” | 269,065 |
| #12 | 9 or 11 | 327,366 |
| #14 | traffic pollution/ | 420 |
| #15 | 10 or 14 | 185,293 |
| #16 | (“air pollution control” or “air quality control” or “ambient air pollution” or “atmospheric pollut*” or “ambient particulate matter” or “ultrafine particle” or “vehicle emissions” or “ low emission zone”).mp. [mp=title, abstract, heading word, drug trade name, original title, device manufacturer, drug manufacturer, device trade name, keyword heading word, floating subheading word, candidate term word] | 26,947 |
| #17 | 15 or 16 | 191,745 |
| #18 | #17 and #12 | 1,820 |

**Cochrane library**

|  | **Search terms** | **No. of hits** |
| --- | --- | --- |
| #1 | MeSH descriptor: [Air pollution] this term only | 137 |
| #2 | MeSH descriptor: [Air pollution, Indoor] explode all trees | 267 |
| #3 | MeSH descriptor: [Particulate Matter] this term only | 212 |
| #4 | MeSH descriptor: [Smog] explode all trees | 3 |
| #5 | MeSH descriptor: [Dust] explode all trees | 290 |
| #6 | MeSH descriptor: [Vehicle Emissions] explode all trees | 90 |
| #7 | MeSH descriptor: [Traffic-Related Pollution] in all MeSH products | 4 |
| #8 | MeSH descriptor: [Cost-Benefit Analysis] explode all trees | 7,791 |
| #9 | (“Air pollut*” OR “air quality” OR “urban pollut*” OR “ambient air pollution” OR “atmospheric pollut*” OR “air contamination” OR “ambient particulate matter” OR “air control” OR “air-pollution”):ti,ab,kw | 1,649 |
| #10 | (“nitrogen dioxide” OR “nitrates” OR “ ozone” OR “O3” OR “NO2” OR “Sulfur Dioxide” OR “SO2”): ti,ab,kw | 6,514 |
| #11 | “cost-effectiveness” OR “cost benefit” OR “ cost minimization” OR “cost-utility” OR “rate of return” OR “net present value” OR “NPV” | 22,393 |
| #12 | “particulate matter” OR “Airborne matter” OR “Air pollutants” OR “PM” OR “UFPM” OR “vehicle emission” OR “low emission zone” | 21,998 |
| #13 | #1 OR #2 OR #3 OR #4 OR #5 OR #6 OR #7 OR #9 OR #10 OR #12 | 29,168 |
| #14 | #11 OR #8 | 22,393 |
| #15 | #14 AND #13 | 344 |

”

**Web of science, CEA registry and Scopus**

(““air pollu*” OR “ambient air pollution” OR “atmospheric pollu*” OR “particulate matter” OR “PM*” OR “air control” OR “air quality” OR “traffic related pollution” OR “vehicle emission” OR “low emission zone) AND (“cost-effectiveness” OR “cost benefit” OR “cost minimization” OR “cost-utility” OR “rate of return” OR “net present value” OR “NPV”)
