# Supplementary material for: The costs, health and economic impact of air pollution control strategies: a systematic review
Source: Glob Health Res Policy. 2024 Aug 21;9:30. doi: 10.1186/s41256-024-00373-y (PMC11337783; doi:10.1186/s41256-024-00373-y)
Supplement: Supplementary file 2 — Additional file 2. [file 41256_2024_373_MOESM2_ESM.docx]

**Data elements of the systematic review**

| **Data elements of the review** |
| --- |
| - Study identification: authors, year of publication - Country(ies) of conduct - Study objective and summary results - Study design:   - Ex post analyses or Ex ante analyses  - Study location and scope (e.g., city/country of conduct; high income setting, low income setting low-and-middle income setting^[[1]](#footnote-1)^, national or sub-national scope)  - Study period: intervention starting and ending date; intervention duration.   - Study methodology:   - Methodologies to estimate costs  - Methodologies to model the reduction in pollution exposure attributable to the control strategy or scenarios considered  - Methodologies to estimate benefits   - Types of air pollution control strategy (e.g., outdoor interventions reducing ambient pollution, indoor interventions reducing household pollution, or mixed interventions) - Pollution control method (e.g., source reduction, which mitigates pollution at its source or end-of-pipe treatments, which mitigates pollution post-formation) - Pollution control strategy categories (e.g., forestry and agricultural measures, transport regulations, health co-benefits of global climate change policies, emission regulation standards/caps, cleaner/alternative energy source, emissions reduction technology, indoor air quality control technology, household clean heating, household cooking strategies) - Particulate matter or hazardous gas considered (PM2.5, PM10, O3, NOX, SOX, NH4, CO). - Emissions modelling (e.g., Global Change Assessment Model, Greenhouse Gas and Air Pollution Interactions and Synergies, Weather Research and Forecasting model) - Types of costs considered (e.g., operating costs, maintenance costs, capital costs) - Mitigation costs estimates - Health endpoints considered (e.g., premature deaths, mortality, morbidity, reduced working days, cancer, etc.) - Health assessment model (e.g., Environmental Benefits Mapping and Analysis Program), and benefits evaluation method (e.g., willingness to pay estimate, cost-of-illness estimate) - Total benefits (including health benefits, or wider assessment of social benefits including benefits to society, environment and ecology) - Cost-benefit analyses results (e.g., Net Present Value (NPV), Net benefits, Benefit-cost ratios or other) - Sensitivity analyses and results (Parameters considered, net present value, net benefits, benefit cost ratios under alternative scenarios) |

1. World bank classification in 2024. Available from: https://datahelpdesk.worldbank.org/knowledgebase/articles/906519-world-bank-country-and-lending-groups [↑](#footnote-ref-1)
