# Supplementary material for: The costs, health and economic impact of air pollution control strategies: a systematic review
Source: Glob Health Res Policy. 2024 Aug 21;9:30. doi: 10.1186/s41256-024-00373-y (PMC11337783; doi:10.1186/s41256-024-00373-y)
Supplement: Supplementary file 4 — Additional file 4. [file 41256_2024_373_MOESM4_ESM.docx]

| **Study ID** | **Country(s) of conduct** | **Indoor or Outdoor intervention** | **Pollution control methodology** | **Typology of pollution control** | **Pollutant** | **Valuation method** | **Health endpoints** | **Social**  **benefit framework** | **Reported Value of control strategy** |
| --- | --- | --- | --- | --- | --- | --- | --- | --- | --- |
| Aldred 2016 (1) | US | Indoor | End of pipe treatment | indoor air ventilation/filters/cleaners/purifiers | O3 | WTP | mortality, Respiratory HA, Dysrhythmia HA, acute respiratory morbidity, School loss day, Respiratory HA | No | Each of the 12 cities has a median B/C value >1.0 for the optimal condition. The mean predicted benefit-to-cost ratios for 1-inch filters were >1.0 in 10 of the 12 cities. |
| Antturi 2016 (2) | Baltic Sea | Outdoor | Multi method (low-sulphur fuel and sulphur scrubber) | Transport regulation (Baltic Sea Sulphur Emission Control Area) | PM2.5 | Not specified | cardiopulmonary disease, lung cancer, chronic obstructive pulmonary disease, restricted activity days | No | B/C ratio: 0.23 |
| Åström 2018 (3) | EU | Outdoor | multi method (low-sulphur fuel and sulphur scrubber) | Transport regulation (nitrogen emission control area) | NOX, PM2.5, SO2, CH4 | VSL (range between 1,218,000–3,130,000 €2010 per avoided fatality) | asthma symptom days (age group 5–19); bronchitis in children (6–12); cardiovascular hospital admissions (18+); chronic bronchitis (27+); lost working days (15–64); respiratory hospital admissions (all ages); and restricted activity days (all ages). | Yes- total benefits containing health, CO2 and crop | B/C ratio of NECA-BAS (Nitrogen Emission Control Area in Baltic Sea): 1.25;  B/C ratio of NECA-NSE (Nitrogen Emission Control Area in North Sea and the English Channel): 4.8;  B/C ratio of NECA-BAS + NSE (Nitrogen Emission Control Area in North Sea and Baltic Sea): 4.38;  B/C ratio of NECA-LNG (LNG propulsion engines on new ships): 10.12 |
| Aunan 1998 (4) | Hungary | Outdoor | multi method (National Energy Efficiency Improvement and Energy Conservation Programs) | Multicategory | TSP, SO2, NO2, dust | WTP | Deaths >65 years, Infant deaths, Lung cancer, ARS-Child-mild, ARS-Child-restricted, ARS-Child-HA, Pseudo-croup, ARS-Adult-mild, ARS-Adult-mild, ARS-Adult-HA, Asthma days adult, CRS-Child, CRS-Adult | Yes- crop loss, Damage to materials, Climate change | The estimated annual benefit of improved health conditions alone is likely to exceed the investments needed to implement the program |
| Aunan 2013 (5) | China | Indoor | Source reduction strategies (cleaner stoves) | household energy strategies | PM2.5 | WTP | COPD among women | NO | B/C ratio of no-chimney stove to Second generation improved stove:14.5;  B/C ratio of chimney stove to Second generation improved stove:3.7 |
| Babcock Jr 1973 (6) | US | Outdoor | multi method | Multicategory | SOX, PM | Not specified | not specified | Yes- Residential property, Materials, Vegetation | B/C ratio: 2.56 |
| Ballini 2015 (7) | Denmark | Outdoor | Source reduction strategies (cold-ironing) | Transport regulation | S02, NOX, PM, CO2 | Not specified | not specified | Yes | capital cost would be recovered by saved health costs in 12–13 years |
| Barstow 2019 (8) | Rwanda | Indoor | Source reduction strategies (cookstoves) | household energy strategies | PM2.5 | Not specified | not specified | Yes- Time Savings, reduced deforestation and reductions in carbon emissions | B/C ratio: 5.6 |
| Beatty 2011 (9) | US | Outdoor | End of pipe treatment (retrofitting diesel oxidation catalysts) | Vehicle emission reduction technology | not specified | COI | bronchitis, asthma, pleurisy, and pneumonia Bronchitis, asthma, and pneumonia outcomes | No | the net present value children’s health benefits are between 424,000 and 989,000 dollars per adopter school district |
| Bollen 2009 (10) | EU, China | Outdoor | Source reduction strategies | Greenhouse Gas policy | PM2.5 | VSL (1.06 million, $US 2000) | short term: eye irritation and chronic bronchitis or asthma, long term: restricted activity days, cancers, and premature deaths | No | Not specifically reported |
| Bonilla  2023 (11) | Colombia | Outdoor | not specified | Multiple (transport regulations, cleaner energy) | PM2.5 | VSL (US $1.72 million) | Premature deaths | Yes (Land and house building values) | NPV of Paving Roads (in USD millions): 6,723.3;  NPV of industrial fuel substitution (in USD millions): 1,743.7;  NPV of reduced vehicle emissions (in USD millions): 584.1 |
| Borjesson 2021 (12) | Sweden | Outdoor | Source reduction strategies (low emission zone) | Transport regulation | NOX4 | VSL (0.13 million, € 2018 prices) | not specified | No | B/C ratio: 0.07 |
| Bouscasse 2022 (13) | France | Mixed | source reduction | Multiple (transport regulations, cleaner heating, cleaner stoves) | PM2.5 | Not specified | Premature deaths, Lung cancer, Stroke, Ischemic heart disease | Yes (increased physical activity, traffic accident risk) | B/C ratio of heating_1 (replace 1/3 of non-efficient wood stove with efficient wood stoves): 26.8;  B/C ratio of heating_2 (replace all non-efficient wood stove with efficient wood stoves): 26.7;  B/C ratio of heating_3 (replace all of non-efficient wood stove with pellet stoves): 35.4;  B/C ratio of traffic_1 (planned LEZ): 67;  B/C ratio of traffic_1 (extended LEZ): 19.8;  B/C ratio of modal shift to public transport:1.1;  B/C ratio of modal shift to active modes: 3.1;  B/C ratio of modal shift to E-bike modes: 4.7, |
| Buonocore 2016 (14) | US | Outdoor | Not specified (carbon standard) | Energy regulation standards/caps | PM2.5,O3 | COI | Mortality-All Cause, Hospital Admission-All Respiratory, Hospital Admission- All Cardiovascular, Acute Myocardial Infarction Non-fata | No | B/C ratio: 1.71 |
| Burtraw 2001 (15) | US | Outdoor | Source reduction strategies | Energy regulation standards/caps | NOX, PM10,SO2 | Not specified | acute morbidity effects of various types, the number of chronic disease cases, and the number of statistical lives lost to premature death. | No | B/C ratio of annual cap on NOX: 0.58 |
| Cai 2018 (16) | China | Outdoor | not specified | Energy regulation standards/caps | primary: SO2, NOX. Secondary: PM2.5 | Not specified | Ischaemic heart disease, Stroke, COPD, Lung cancer | No | B/C ratio: 3-9 |
| Carnevale 2018 (17) | Italy | Mixed | Multi method (PRIA Air Quality Plan) | Multicategory (domestic heating, transport, energy and agriculture regulations) | PM10 | COI | mortality, simple cough and restricted activity days (RADs), cerebrovascular problems, chronic bronchitis | Yes- Direct benefits from reduced energy uses, reduced greenhouse gas emission | B/C ratio: 1.78 |
| Ćetković 2020 (18) | Montenegro | Mixed | Multi method | Multicategory | Not specified | Not specified | not specified | No | B/C ratio of flue desulphurization system:5.91;  B/C ratio of flue denitrification system:1.52;  B/C ratio of alternative fuels use, new vehicle generations:0.01;  B/C ratio of heating devices replacement:0.29;  B/C ratio of long-distance heating system: 2.52;  B/C ratio of agricultural regulations: 3.23;  B/C ratio of organic agriculture production and animal waste management system improvement: 0.34;  B/C ratio of ammonia emissions control:1.11;  B/C ratio of prohibition of ammonium carbonate agents: 1.11 |
| Chau 2008 (19) | China, Hong Kong | Indoor | End of pipe treatment (indoor air cleaners) | Indoor air ventilation/filters/cleaners/purifiers | PM10 | COI, HC, WTP | Hospital admissions: Respiratory diseases (adult and elderly), cardiovascular diseases (adult and elderly). Mortality: Respiratory diseases (adult and elderly), cardiovascular diseases (adult and elderly). Restricted activity day | No | B/C ratio for adults’ whole year: 0.61;  B/C ratio for adults’ cool seasons only: 0.61;  B/C ratio for elderly (aged 65+) whole year: 1.01;  B/C ratio for elderly (aged 65+) cool seasons: 1.41; |
| Chen 2015 (20) | East Asia | Outdoor | not specified | not specified | O3, PM2.5 | WTP, HC | Premature mortality | No | B/C ratio of strict policy implementation case: 8.2-22;  B/C ratio of less strict policy case: 22- 61 |
| Chen  2022 (21) | China | Outdoor | source reduction  (combined heat and power systems) | cleaner energy | PM2.5 | VSL | Premature Death, Respiratory Diseases hospitalization, cardiovascular diseases hospitalization, acute and chronic Bronchitis, Asthma | Yes (CO2, cost Reduction due to the use of oil associated petroleum gas) | B/C ratio: 2.75 |
| Chestnut 2006 (22) | US | Outdoor | not specified | not specified | primary: NOX, VOC, SO2. secondary: PM2.5, O3 | WTP with minor adjustments for differences in the timing of the risk reductions | mortality and morbidity | No | B/C ratio:17.72 |
| Cropper 2019 (23) | India | Outdoor | End of pipe treatment (retrofitting flue-gas desulfurization unit) | point emission reduction technology | primary S02, secondary PM2.5 | WTP extrapolated from high income countries. | Mortality | No | NPV ranged from -243 million to 6650 million from Tuticorin plant to Dadri plant (2015 PPP$) |
| Evans 2021 (24) | Mexico | Outdoor | End of pipe treatment (particle filter) | Vehicle emission reduction technology | PM2.5 | VSL results extrapolated from US | ischemic heart disease (IHD), cerebrovascular stroke (STK), chronic obstructive pulmonary disease (COPD), and trachea, bronchus, and lung cancers (LC), acute lower respiratory infections (children) | No | Not specifically reported |
| Feng 2021 (25) | China | Indoor | Source reduction | Clean heating | PM2.5 | COI from the China Health and Family Planning Yearbook 2007–2015 | cardiovascular diseases, cerebrovascular diseases and respiratory diseases, all-cause premature deaths | No | For all regions, the total health economic benefits are larger than the total costs with net benefits of 35.2 million. The cost-benefit ratio of Beijing and Tianjin are 1:1.43 and 1:1.10, respectively. |
| Fisk 2017 (26) | US | Indoor | End of pipe treatment | Indoor air filters | PM2.5 | not specified | not specified | No | Economic benefits always exceed costs with benefit-to-cost ratios ranging from approximately 3.9 to 133 |
| Fisk 2017 (27) | US | Indoor | End of pipe treatment | Indoor air cleaners | PM2.5 | COI based on the medical care consumer price index | All respiratory, Asthma, Acute bronchitis or bronchiolitis, COPD, Pneumonia | No | For the interventions with portable air cleaner use, mortality-related economic benefits exceed intervention costs |
| Fung 2019 (28) | China | Outdoor | Source reduction (intercropping) | Forestry and agricultural measures | primary: NH3, secondary: PM2.5 | not specified | not specified | Yes- revenue from grain yields, saved cost from fertilizer | Net economic benefit of US$67 billion, of which US$13 billion arises from saved health costs from reduced air pollution. |
| Giannakis 2019 (29) | EU | Outdoor | Multi method (improving urea fertilizer application, covered manure storage, low nitrogen feed, low emission animal housing) | Forestry and agricultural measures | primary: NH4, secondary:PM2.5 | WTP based on US VSL of 3 million USD | ischaemic heart disease, cerebrovascular disease, lower respiratory tract infections, chronic obstructive pulmonary disease and lung cancer | No | B/C ratio of low nitrogen feed for EU-28: 169.13;  B/C ratio of covered manure storage for EU-28: 57.14;  B/C ratio of improving urea fertilizer application for EU-28: 53.36;  B/C ratio of low-emission animal housing for EU-28: 3.61; |
| Guo 2018 (30) | China | Mixed | Source reduction (coal cap policy) | Emission standards/limits | PM2.5 | WTP, COI, HC | premature deaths, hospital admissions because of respiratory system and cardio vascular system diseases, outpatient visits of internal medicine and paediatrics, chronic bronchitis, acute bronchitis, and asthma disease | No | B/C ratio: 1.92 |
| Guo 2022 (31) | China | Indoor | Source reduction | Clean/improved heating | PM2.5 | VSL | Morbidity: Respiratory hospital admissions, Cerebrovascular hospital admission, Cardiovascular hospital admissions, Chronic bronchitis, Asthma attacks, Work loss day, Respiratory symptoms day. Mortality: All cause, Chronic obstructive pulmonary disease, Lung cancer, Ischemic heart disease, Stroke, Acute lower respiratory infections | No | B/C ratio of coal-to-gas: 0.38;  B/C ratio of coal-to-electricity: 0.51;  B/C ratio of industrial surplus heat: 0.41;  B/C ratio of current clean heating strategy: 0.45;  B/C ratio of current improved heating strategy: 0.70 |
| Gupta 2021 (32) | India | Indoor | Source reduction strategies (cookstoves) | Household energy strategies | PM2.5, CO | COI | not specified | Yes | B/C ratio: from 3.2 to 4 |
| Howard 2019 (33) | China | Outdoor | not specified | Emission standards/limits | PM10 | VSL for 2015 of $10 million adjusted for Brazil based on purchasing power | Mortality: Cerebrovascular disease, Chronic obstructive pulmonary disease, Ischemic heart disease, Lung cancer, Lower respiratory infection, All causes (infants). Hospital admissions: All cardiovascular, Asthma, Chronic lung disease, All respiratory | No | B/C ratio of cutting PM10 emission from 28.15 g/kWh to 0.69 g/kWh: 60;  B/C ratio of cutting PM10 emission from 0.69 g/kWh to 0.36 g/kWh: 103;  B/C ratio of cutting PM10 emission from 0.36 g/ kWh to 0.04 g/kWh: 89; |
| Hsieh 2022 (34) | China | Outdoor | Source reduction strategies (Electric vehicle) | Transport regulations | primary: CO2, CO, VOC, NOX, SO2, primary PM2.5, secondary: O3, PM2.5 | WTP, COI | premature deaths, hospital admission due to respiratory disease, hospital admission due to cardiovascular disease, chronic bronchitis, asthma attack, emergency room visits for respiratory disease | No | B/C ratio of: 0.76 |
| Huang 2020 (35) | China | Outdoor | Not specified | Not specified | primary: NOx, VOCs, PM, SO2 and NH3. secondary: PM2.5, O3 | not specified | not specified | No | B/C ratio of obtaining PM2.5 (<35 μg/m^3 ) and O3 (<80 ppb): 17.7 |
| Hutchinson 2004 (36) | UK | Outdoor | End of pipe treatment (Vehicle exhaust catalysts) | Vehicle emission reduction technology | Primary: PM10, NOX, VOCs, CO, Secondary: O3, PM10 | VSL of 2,260,000 ($US 1998), WTP, COI | Mortality | No | NPV by 1998: £500 million;  NPV by 2005: £2 billion, |
| Hutton 2007 (37) | World | Indoor | Source reduction strategies (clean stoves) | Household energy strategies | not specified | Health care savings: COI, productivity loss: Human capital | acute lower respiratory infections (ALRI) in children younger than 5 years; and chronic obstructive pulmonary diseas e (COPD) and lung cancer in women and men older than 30 years. | Yes- time savings, environmental greenhouse gas reduction, | B/C ratio for liquefied petroleum gas: 6.9  B/C ratio for improved stove: negative (A negative benefit-cost ratio means that intervention cost savings exceed intervention costs. Net costs are negative.) |
| Irfan 2021 (38) | Pakistan | Indoor | Source reduction strategies (LPG, natural gas, biogas, electric stoves, ICS) | Household energy strategies | not specified | converted the estimated VSL 0.248 million USD into PKR 21.45 million | not specified | Yes- Fuel saving, time saving, productivity gain | B/C ratio for biogas: 2.54;  B/C ratio for LPG: 4.64;  B/C ratio for Natural gas: 4.64;  B/C ratio for electric stove: 3.07;  B/C ratio for ICS: 0.38; |
| Isihak 2012 (39) | Nigeria | Indoor | Source reduction strategies | Household energy strategies | not specified | COI | not specified | Yes- Productivity, Time savings, greenhouse gas reduction | B/C ratio of stove: 2.58;  B/C ratio of LPG: 2.70 |
| Iwata 2011 (40) | Japan | Outdoor | End of pipe (diesel particulate filters) | Vehicle emission reduction technology | NOX | not specified | not specified | No | B/C ratio: 7.31 |
| Jeuland 2018 (41) | not specified | Indoor | Source reduction strategies | Household energy strategies | not specified | not specified | acute lower respiratory illness, chronic obstructive pulmonary disease, lung cancer and ischemic heart disease | Yes - forest benefit, CO2 emission savings, health, time saving, net fuel savings | B/C ratio of ND biomass: 1.69;  B/C ratio of FD biomass: 3.61;  B/C ratio of charcoal: 7.6;  B/C ratio of kerosene: 0.51;  B/C ratio of LPG: 1.22;  B/C ratio of electric stove: 4.46 |
| Jin 2017 (42) | China | Mixed | Source reduction strategies | Multiple | PM2.5, SO2, NOX | not specified | ALRI, COPD, Lung cancer | No | B/C ratio of coal-to-gas: 0.23;  B/C ratio of thermal insulation: negative (intervention cost savings exceed intervention costs);  B/C ratio of electric stove: 1.86  B/C ratio of thermal insulation and electric stove: 5.6 |
| Kiely 2021 (43) | Indonesia | Outdoor | Prevention strategy | forestry and agriculture measures | PM2.5 | not specified | premature deaths | Yes | B/C ratio:2.27 |
| Kim 2020 (44) | South Korea | Outdoor | mixed methods | Greenhouse Gas policy | PM2.5, O3 | WTP | all cause: work loss days, Respiratory hospital admissions, Cerebrovascular hospital admissions, Cardiovascular hospital admissions, Chronic bronchitis, Asthma attacks, Respiratory symptoms days. Bronchodilator usage, Lower respiratory symptoms, Consultation for allergic rhinitis | No | B/C ratio of SSP2 in 2050: 2.85;  B/C ratio of SSP3 in 2050: 1.80; |
| Kiziltan 2022 (45) | Turkey | Outdoor | Source reduction (cash-for-replacement, emission tax) | Transport regulations | Not specified | not specified | Not specified | Yes | B/C ratio of SCE-1 (3% unregistered car share, 25% gasoline, 27% diesel, 48% LGP by 2030):1.92;  B/C ratio of SCE-2 (1.5% unregistered car share, 25% gasoline, 27% diesel, 48% LGP by 2030): 1.92;  B/C ratio of SCE-3 (3% unregistered car share, 22% gasoline, 14% diesel, 48% LGP by 2030): 1.21;  B/C ratio of SCE-4 (3% unregistered car share, 19% gasoline, 13% diesel, 48% LGP, 16% Hybrid, 4% EV by 2030): 0.87 |
| Krewitt 1999 (46) | EU | Outdoor | not specified | Emission standards/limits | primary: SO2, NOx, NH3, VOC, secondary: sulphate and nitrate aerosols, ozone, PM10. | mortality: value of a life year lost (VLYL), morbidity: WTP | Mortality: Respiratory hospital admissions, Restricted activity days, Cardiovascular hospital admissions, chronic bronchitis, chronic cough, asthmatics, Lower respiratory symptoms, Asthma attacks | Yes – Crop gains, reduction in material damages | B/C ratio of reducing emission level within each EMEP grid by 50%: 13.03;  B/C ratio of joint optimization for acidification and ozone: 11.60;  B/C ratio of maximum feasible reduction: 4.62 |
| Lai  2020 (47) | Taiwan, China | Outdoor | Multiple | Multiple | PM2.5, SOX, NOX, VOC | VSL of 357.9 million NTD in 2014 | Premature deaths | No | B/C ratio of retire diesel trucks: 7.5;  B/C ratio of ships regulation: 332.4;  B/C ratio of power regulation: 144.9;  B/C ratio of restaurant smoke control 183.1;  B/C ratio of motorcycle retirement: 3.2;  B/C ratio of 90% reduction in open burning: 179.3;  B/C ratio of cleaner boilers: 0.8;  B/C ratio of exhaust filters: 7.8;  B/C ratio of pollution control device: 1.1;  B/C ratio of centralized burning: 9.8 |
| Lange 2018 (48) | US | Outdoor | not specified | Emission standards/limits | O3, PM, CO, NO2, SO2 | not specified | not specified | No | Net benefit of between −$0.3 and $1.8 billion for a 0.070 ppm standard and between −$23 and −$17 billion for a 0.065 ppm standard |
| Larson 1999 (49) | Russia | Outdoor | not specified | Emission standards/limits | PM10 | WTP | Mortality | No | Net benefit of $40 million |
| Lavee 2018 (50) | Israel | Outdoor | not specified | Emission standards/limits | primary: PM2.5, PM10, secondary: O3 | WTP, COI, HC and VOLY | PM2.5: Overall mortality, Lung cancer mortality, Mortality from ischemic heart disease. PM10: Overall mortality, Hospitalizations (heart and vascular diseases), Hospitalizations (respiratory diseases), Asthma (Use of medications). O3: Mortality, Hospitalizations (respiratory diseases),Hospitalizations (respiratory diseases) | No | Net total benefit of 949 million NIS (energy sector: 87.8 NIS, industry sector: 523.3 NIS, transport sector: 338 NIS) |
| Levy 2017 (51) | US | Outdoor | End of pipe treatment (pellet boiler with electrostatic precipitator) | Point emission reduction technology | PM2.5 | WTP, COI | premature mortality, acute myocardial infarctions (heart attacks), respiratory and cardiovascular hospital admissions, minor restricted activity days, and lower respiratory symptoms. | No | B/C ratio: 9.7 |
| Li 2004 (52) | China | Outdoor | Source reduction (energy sector cleaner coal combustion technology and urban industrial coal ban): | Cleaner or alternative energy sources | PM10 | WTP, COI | acute mortality, chronic pulmonary disease, hospital visits, and emergency room visits | No | B/C for power sector initiative 1.06;  B/C for industry sector initiative 2.83; |
| Li 2011 (53) | Thailand | Outdoor | Source reduction (vehicle I/M programs) | Transport regulations | PM2.5, PM10, TSP | WTP, COI | Chronic bronchitis, Respiratory hospital admissions, Cardiovascular hospital admissions, Emergency room visits, Acute asthma attacks, Acute respiratory symptom days, Restricted activity days, Mortality | No | B/C ratio: 10.09 |
| Liu 2021 (54) | China | Indoor | End of pipe treatment | Indoor air purifier | PM2.5 | MVSL (marginal increase of VSL) for Chongqing adjusted for different per capita income | ischemic heart disease (IHD), stroke, chronic obstructive pulmonary disease (COPD), lung cancer (LC), and lower respiratory infections (LRI) | No | B/C ratio of 35ug/m^3 PM concentration: 2.61;  B/C ratio of 25ug/m^3 PM concentration: 1.52; |
| Lomas  2016 (55) | UK | Outdoor | Source reduction  (Low emission zones) | Transport regulations | PM, NO2 | COI | All-cause mortality, Coronary events, asthma, low birth weight, Preterm birth | No | B/C ratio: 0.85 |
| Lopez 2020 (56) | Philippines | Outdoor | Source reduction | Transport regulations | NOX, PM, SO2, VOC | not specified | not specified | No | most alternatives are clustered around the −0.03 to +0.01, and −0.0001 to +0.0004 ranges for net ownership savings and net societal benefits, respectively. |
| Lopez-Aparicio 2020 (57) | Norway | Outdoor | Source reduction (speeding limit) | Transport regulations | PM10, NOX | not specified | mortality: cardiovascular and lung disease. | Yes – reduced noise exposure and traffic accidents | B/C ratio of current trends in drive compliance with the speed limit:1.24;  B/C ratio of full compliance with the speed limit: 0.79 |
| Luo 2022 (58) | US | Outdoor | Not specified | Greenhouse Gas policy | PM2.5 | not specified | not specified | No | Not specifically reported |
| Malla 2011 (59) | Nepal, Kenya and Sudan | Indoor | Source reduction | Household energy strategies | CO | COI | morbidity of COPD for men and women over 30 and morbidity of ALRI in children under 5 years | Yes - Fuel savings, cooking time savings | B/C ratio in Nepal: 1.4;  B/C ratio in Kenya: 21.4;  B/C ratio in Sudan: 2.5 |
| Mao 2005 (60) | China | Outdoor | Source reduction (coal-to-natural gas) | Cleaner/alternative energy sources | SO2, TSP, secondary: PM10. | not specified | not specified | No | NPV of Chongqing project: 18,649,523 (1998 RMB);  NPV of Beijing project: 6,876,035 (1998 RMB) |
| Mardones 2021 (61) | Chile | Indoor | Source reduction (heater replacement program) | Clean heating | PM2.5 | VSL of US $ 3.7 million | premature mortality | No | B/C ratio of wood-burning heater: 0;  B/C ratio of kerosene: 2.5;  B/C ratio of pellet heater: 2.13 |
| Markandya 2018 (62) | World | Outdoor | not specified | Greenhouse Gas policy | organic carbon, black carbon, NOX, non-methane, CO, and SO2, secondary: PM2.5, O3 | VSL ranging between US$1·8 and $4.5 million | for ozone coverage is for respiratory disease and for PM2.5 it is for ischaemic heart disease, chronic obstructive pulmonary disease, stroke, lung cancer, and acute lower respiratory airway infections | No | The ratio of health co-benefit to mitigation cost ranged from 1.4 to 2.45 |
| Mazorra  2020 (63) | Senegal, The Gambia, Guinea Bissau | Indoor | Source reduction | Clean cooking | PM2.5 | not specified | COPD, ALRI | Yes (time savings, fuelwood savings) | NPV ICS life span 2 years (in millions of euros): 13.87;  NPV ICS life span 4 years (in millions of euros): 14.76;  NPV ICS life span 5 years (in millions of euros): 14.93 |
| Mesbah 2013 (64) | US | Outdoor | Source reduction | Emission standards/limits | primary: NOX, secondary: O3 | VSL- $6.8 million in 2007 | not specified | No | B/C ratio of damage minimization policy: 2.29;  B/C ratio of social cost minimization policy: 2.93;  B/C ratio of abatement costs minimization policy: 18.29 |
| Miraglia 2007 (65) | Brazil | Outdoor | Source reduction (diesel/ethanol fuel mixture) | Cleaner/alternative energy sources | PM10, NOX, CO | VSL | paediatric respiratory hospital admissions, elderly respiratory hospital admissions, emergency room visits due to ischemic cardiovascular diseases, all-cause elderly mortality, elderly respiratory disease mortality, elderly cardiovascular mortality | Yes | B/C ratio: 1.09 |
| Miranda 2016 (66) | Portugal | Mixed | mixed methods | Multicategory | PM10, NO2 | not specified | PM10: asthma, heart failure, chronic bronchitis, total mortality. NO2: respiratory hospital admissions, total mortality. | No | B/C ratio of replace 10% of vehicles below the EURO3 class with hybrid vehicles (HYB):0.75;  B/C ratio of residential combustion regulations (FIR):2.25;  B/C ratio of low emission zone (LEZ):1.03;  B/C ratio of 10% clean combustion technology in industry sector (IND): 0.97;  B/C ratio of HYB + FIR: 1.18;  B/C ratio of FIR + IND: 1.14;  B/C ratio of HYB + FIR + LEZ + IND: 1.03 |
| Moon 2021 (67) | South Korea | Outdoor | Not specified (The 2nd Comprehensive Plan for Air Quality Improvement) | Emission standards/limits | NOX, PM2.5, PM10, VOC, fine dust | WTP | not specified | No | B/C ratio: 0.61 |
| Netalieva 2005 (68) | Kazakhstan | Outdoor | Source reduction (fines for pollution which exceeds the maximum permissible concentration) | Emission standards/limits/policy | not specified | not specified | Runny nose, Headache, Fever, Cough, Irritated eyes | No | B/C ratio: 5 |
| Nishioka 2005 (69) | US | Indoor | Source reduction (insulation) | Clean/improved heating | PM2.5, S02, NOX | not specified | not specified | Yes - direct cost savings from reduced energy reductions | NPV: $500 million |
| Nuhu 2022 (70) | Ghana | Indoor | Source reduction (improved cooking) | Household energy strategies | not specified | COI | Acute Lower Respiratory Illness (ALRI), ischemic heart disease (IHD), lung cancer and Chronic Obstructive Pulmonary Disease (COPD) | Yes | B/C ratio of ICS charcoal: 0.67;  B/C ratio of ICS wood: 0.47;  B/C ratio of LGP: 0.55; |
| Okada 2019 (71) | not specified | Outdoor | Source reduction (emission control area) | Transport regulations | SO2 | VSL of 20,000 | Mortality | No | Not specifically reported |
| Olsthoorn 1999 (72) | EU | Outdoor | Not specified | Emission standards/limits/policy | SO2, NOX, PM10 | VOSL estimate of 3.2 million ECU; WTP using a value of 2.6–4.2 million ECU | Mortality, Hospital emergency room admissions, Upper respiratory symptoms children, Lower respiratory symptoms children, Restricted activity days, Respiratory morbidity children, Respiratory symptom prevalence | Yes - material damages | In the case of PM10 the benefits exceed the costs by a factor 100 to 200 and that for SO2 and NO2 the benefit cost ratios are in the range of 1–10. |
| Ou 2020 (73) | US | Outdoor | US | Emission standards/limits/policy | PM2.5, SO2, NOX | not specified | not specified | No | B/C ratio of 10% reduction relative to current base trajectory by 2050: 102.1;  B/C ratio of 20% reduction relative to current base trajectory by 2050: 29.8;  B/C ratio of 30% reduction relative to current base trajectory by 2050: 13.6;  B/C ratio of 40% reduction relative to current base trajectory by 2050: 6.2;  B/C ratio of 50% reduction relative to current base trajectory by 2050: 2.2; |
| Palmer 2007 (74) | US | Outdoor | Not specified (Clean Air Interstate Rule, CAIR) | Emission standards/limits/policy | PM and ozone and mercury | not specified | not specified | No | B/C ratio of CAIR with mercury cap: 2.44;  B/C ratio of CAIR with mercury cap and seasonal state implementation NOX plan: 2.17;  B/C ratio of CAIR plus maximum achievable control technology (MACT) approach: 6.58;  B/C ratio of: CAIR plus mercury cap-and-trade: negative |
| Pandey 2003 (75) | Canada | Outdoor | not specified | Emission standards/limits/policy | PM, O3 | human capital, WTP | not specified | No | B/C ratio: 3 |
| Perl 1982 (76) | US | Outdoor | Source reduction (State implementation plan, new source performance standards) | Emission standards/limits/policy | SO2 | not specified | cardiovascular and respiratory disease | No | benefits exceeded costs for SO2 controls imposed under the 1970 Clean Air Act, but 1979 revisions generated costs well in excess of benefits |
| Pisoni 2009 (77) | Italy | Outdoor | not specified | not specified | PM10 | COI | Respiratory Hospital Admissions (RHA), the Cerebrovascular Hospital Admissions (CVA) and the Years Of Lost Life (YOLL) | No | Not specifically reported |
| Raff 2020 (78) | US | Outdoor | not specified (National Ambient Air Quality Standards) | Emission standards/limits/policy | SO2, NO2 | not specified | not specified | No | B/C ratio of 10 ug/m3, 25 ug/m3 24-hour mean, 12 plants entering non-attainment: 2.20;  B/C ratio of 8 ug/m3, 25 ug/m3 24-hour mean, 53 plants entering non-attainment: 2.04;  B/C ratio of 8 ug/m3, 25 ug/m3 24-hour mean, 61 plants entering non-attainment: 2.04; |
| Rezazadeh 2022 (79) | Iran | Mixed | Multi method | Multicategory | PM2.5, SO2, and NOx | WTP | not specified | No | NPV of new gas heaters for all houses: $8 million;  NPV of new gas heaters for 30% houses: $1.9 million;  NPV of reducing 10% industry production: - $46.3 million;  NPV of reducing 3% industry production: -$13.8 million;  NPV of converting 30% gasoline cars to CNG-burning cars: $6.9 million;  NPV of converting 20% gasoline cars to CNG-burning cars: $4.6 million;  NPV of vehicle inspection for all: $1.3 million;  NPV of vehicle inspection for 20%: $0.3 million;  NPV of improved fuel consumption for 30% of cars: $1.8 million;  NPV of improved fuel consumption for 20% of cars: $0.7 million;  NPV of improved fuel consumption for 20% of cars: $0.7 million;  NPV of car rideshare (all class 1 cars): $1.8 million;  NPV of car rideshare (20% class 1 cars): $0.7 million |
| Sampedro 2020 (80) | World | Outdoor | not specified | Greenhouse Gas policy | PM2.5, O3 | WTP | Ischemic heart disease (IHD), chronic obstructive pulmonary disease (COPD), stroke, lung cancer (LC), and acute lower respiratory infection (ALRI) | No | B/C ratio from 1.45 to 2.19 |
| Schucht 2015 (81) | EU | Outdoor | not specified | Greenhouse Gas policy | PM2.5, O3 | WTP | Acute Mortality, Respiratory Hospital Admissions, Minor Restricted Activity Days, Chronic Mortality, Infant Mortality, Chronic Bronchitis, Respiratory Hospital Admission, Cardiac Hospital Admission, Restricted Activity Days, Lower respiratory symptom (LRS) days | No | B/C ratio: 0.95 |
| Shindell 2021 (82) | US | Outdoor | not specified | Greenhouse Gas policy | PM, O3 | not specified | premature deaths | Yes - Social cost of carbon | B/C ratio at 2030: 25;  B/C ratio at 2060: 30 |
| Stevens 2005 (83) | Mexico | Outdoor | end of pipe treatment (diesel particulate filters, or diesel oxidation catalysts) | vehicle emission reduction technology | PM2.5 | WTP: A plausible range for a Mexican VSL was obtained by extrapolating from U.S. estimates using income elasticities of 0.5 and 2. | cardiopulmonary, lung cancer mortality, respiratory deaths children, sudden infant death syndrome | No | B/C ratio of particle filter for bus (old model vs new model): 8.1, 1.4  B/C ratio of particle filter for truck (old model vs new model): 2.5, 1.0;  B/C ratio of particle filter for tractor (old model vs new model): 1.9, 0.1;  B/C ratio of oxidation catalyst for bus (old model vs new model): 2.6, 0.7  B/C ratio of oxidation catalyst for truck (old model vs new model): 0.9, 0.4;  B/C ratio of oxidation catalyst for tractor (old model vs new model): 0.7, 0.1; |
| Suhyoung 2021 (84) | Korea | Outdoor | not specified (The 2nd Basic Plan for Seoul Metropolitan Area Air Quality 2014 (BP2014), Master Plan for PM2.5 (MP2019)) | Emission standards/limits/policy | PM2.5 | VSL | not specified | No | B/C ratio of BP2014: 1.2;  B/C ratio of MP2019: 2.07 |
| Tang 2022 (85) | China | Outdoor | Not specified | Greenhouse Gas policy | C02, NH3, NO2, SO2, PM2.5 | not specified | premature deaths | No | Under the 2 °C target, carbon mitigation costs could be more than offset by health co-benefits in 2050, bringing a net benefit of $393–$3,017 billion (in 2017 USD value) |
| Thanh 2001 (86) | Thailand | Outdoor | end of pipe treatment (flue gas desulfurization) | point emission reduction technology | PM10, SO2 | WTP | Premature Mortality, Respiratory hospital admission, Cardiac hospital admission, Days with acute respiratory Symptoms | No | B/C ratio: 2.5 |
| Tse 2004 (87) | Hong Kong, China | Indoor | End of pipe treatment | Air cleaners | PM2.5 PM10 | premature mortality: WTP-upper bound, hedonic wage-lower bound. Hospital admissions: WTP-upper bound, COI-lower bound. | Circulatory system, Respiratory system, All causes mortality, All causes, Pneumonia, COPD, Ischemic heart disease, restricted activity days | No | B/C ratio range from 7.5 to 30.54 |
| Vandyck  2018 (88) | World | Outdoor | not specified | Greenhouse Gas policy | SO2, NOX, PM2.5, CO, organic carbon | VSL- 1.5 million US$(2005) | mortality, cardiovascular and respiratory diseases and lung cancer | Yes (crop yield) | not specified |
| Voorhees 2000 (89) | Japan | Outdoor | mixed methods | Multicategory | NO2 | COI, human capital | phlegm and sputum, eye irritation and sore throat, lower respiratory symptoms | Yes | B/C ratio: 6 |
| Voorhees 2008 (90) | Japan | Outdoor | Not specified | Emission standards/limits/policy | PM | not specified | not specified | No | B/C ratio: 1.8 |
| Wagner 2015 (91) | Germany | Outdoor | mixed methods | forestry and agriculture measures | PM2.5, PM10 | not specified | mixed methods | Yes | B/C ratio of low protein feeding: 0.8;  B/C ratio of Manure storage cover: 7.6, 2.4, 5.2 (for Granulates cover, Swimming foil, Concrete cover);  B/C ratio of Trailing hose: 0.9;  B/C ratio of Trailing shoes: 3.2;  B/C ratio of Injection/cultivator: 3.9;  B/C ratio of air purifier: 4.8;  B/C ratio of biofilter: 2.2  B/C ratio of urban substitution: 8.1, 8.4 (for Baden-Württemberg and Brandenburg) |
| Wagner 2017 (92) | Germany | Outdoor | Source reduction | forestry and agriculture measures | Primary gas: NH3, secondary gas: PM2.5 | WTP and COI | Mortality, chronic bronchitis in adults, restricted activity days, hospital admission | Yes – biodiversity damage cost reduction, | B/C ratio of floating plastic cover and trailing shoe: 3.6;  B/C ratio of floating plastic cover and trailing shoe: 4.2; |
| West 2006 (93) | World | Outdoor | not specified | Emission standards/limits/policy | primary: CH4, secondary: O3 | VSL of $1 million | chronic respiratory conditions, premature mortality, hospital admissions | No | B/C ratio: 2.4 |
| Wiser 2020 (94) | US | Outdoor | Source reduction | cleaner/alternative energy sources | not specified | not specified | not specified | Yes – energy savings | B/C ratio: 38 |
| Wu 2017 (95) | China | Outdoor | not specified | Greenhouse Gas policy | PM2.5 | not specified | chronic bronchitis, upper respiratory infections, asthma, chronic bronchitis | No | B/C ratio of INDC1 scenario: 2.7;  B/C ratio of INDC2 scenario: 1.35;  B/C ratio of YRD scenario: 2.1; |
| Xie 2016 (96) | China | Outdoor | not specified | Emission standards/limits/policy | SO2 | not specified | premature deaths from cardiovascular disease, premature deaths from respiratory disease | No | B/C ratio of cooperative reduction model: 0.28;  B/C ratio of non-cooperative reduction model: 0.25; |
| Yang  2018 (97) | China | Outdoor | Mixed methods | Multiple | SO2, PM2.5 | not specified | premature deaths | No | not specified |
| Zhang 2015 (98) | China | Outdoor | end of pipe treatment | point emission reduction technology | SO2, NOX | COI, WTP | chronic bronchitis, total mortality, restricted activity days, respiratory hospital admissions, cardiovascular hospital admissions | No | B/C ratio of gradual control strategy: 32.10;  B/C ratio of multi pollutant control strategy: 28.95; |
| Zhang 2019 (99) | China | Outdoor | Multiple | Multiple | PM, SOX, NOX | WTP, COI | respiratory and cardiovascular diseases hospitalisations, premature deaths, reduced working days, | Yes- reduced agricultural loss, building material and cleaning cost | Net benefit: 818.0 billion RMB |
| Zhang  2021 (100) | China | Outdoor | Multiple | Alternative energy, Negative emissions technology | PM2.5 | a normal distribution for VSL with a mean of 100 times of annual earnings in China | premature mortality: chronic obstructive pulmonary disease, ischaemic heart disease, stroke, and lung cancer. Hospital admission: respiratory disease, cardiovascular and cerebrovascular disease, ER visits, bronchitis | No | not specified |
| Zhao 2021 (101) | China | Indoor | Source reduction | clean/improved heating | PM2.5 | not specified | mortality | No | B/C ratio for completed renovation: 1.51;  B/C ratio for natural gas renovation: 1.54;  B/C ratio for electric renovation: 1.6; |
| Zhao 2022 (102) | China | Mixed | mixed methods | Multicategory | PM2.5, PM10, SO2, NO2, CO and O3 | not specified | all-cause, respiratory, cardiopulmonary and lung cancer mortalities, and corresponding working time loss | No | B/C ratio: 0.69 |
| Zhou 2019 (103) | China | Outdoor | Source reduction (yellow-label vehicles scrappage subsidy policy) | Transport regulation | PM2.5 | premature deaths: HC, respiratory and cardiovascular diseases: COI | All-cause mortality (mortality associated with chronic effects, mortality associated with acute effects, respiratory diseases, cardiovascular diseases and chronic bronchitis), hospitalization and sick leave | No | There were six cities in the BTH region whose net benefits were greater than zero (Beijing highest), seven cities, the net benefits were less than zero. |
| Zhou  2022 (104) | China | Outdoor | Source reduction | Transport regulations | PM2.5 | Not specified | premature deaths, respiratory diseases, cardiovascular diseases, chronic bronchitis | No | Total net benefit for 13 cities: 92.69 billion CNY |

**Reference**

1. Aldred JR, Darling E, Morrison G, Siegel J, Corsi RL. Benefit-cost analysis of commercially available activated carbon filters for indoor ozone removal in single-family homes. Indoor Air. 2016;26(3):501-12.

2. Antturi J, Hänninen O, Jalkanen JP, Johansson L, Prank M, Sofiev M, et al. Costs and benefits of low-sulphur fuel standard for Baltic Sea shipping. Journal of Environmental Management. 2016;184:431-40.

3. Åström S, Yaramenka K, Winnes H, Fridell E, Holland M. The costs and benefits of a nitrogen emission control area in the Baltic and North Seas. Transportation Research Part D: Transport and Environment. 2018;59:223-36.

4. Aunan K, Pátzay G, Asbjørn Aaheim H, Martin Seip H. Health and environmental benefits from air pollution reductions in Hungary. Science of the Total Environment. 1998;212(2):245-68.

5. Aunan K, Alnes LWH, Berger J, Dong Z, Ma L, Mestl HES, et al. Upgrading to cleaner household stoves and reducing chronic obstructive pulmonary disease among women in rural china - a cost-benefit analysis. Energy for Sustainable Development. 2013;17(5):489-96.

6. Babcock Jr LR, Nagda NL. Cost effectiveness of emission control. Journal of the Air Pollution Control Association. 1973;23(3):173-9.

7. Ballini F, Bozzo R. Air pollution from ships in ports: The socio-economic benefit of cold-ironing technology. Research in Transportation Business and Management. 2015;17:92-8.

8. Barstow C, Bluffstone R, Silon K, Linden K, Thomas E. A cost-benefit analysis of livelihood, environmental and health benefits of a large scale water filter and cookstove distribution in Rwanda. Development Engineering. 2019;4.

9. Beatty TKM, Shimshack JP. School buses, diesel emissions, and respiratory health. Journal of Health Economics. 2011;30(5):987-99.

10. Bollen J, van der Zwaan B, Brink C, Eerens H. Local air pollution and global climate change: A combined cost-benefit analysis. Resource and Energy Economics. 2009;31(3):161-81.

11. Bonilla JA, Aravena C, Morales-Betancourt R. Assessing Multiple Inequalities and Air Pollution Abatement Policies. Environmental & Resource Economics. 2023;84(3):695-727.

12. Borjesson M, Bastian A, Eliasson J. The Economics of Low Emission Zones. Transportation Research: Part A: Policy and Practice. 2021;153:99-114.

13. Bouscasse H, Gabet S, Kerneis G, Provent A, Rieux C, Ben Salem N, et al. Designing local air pollution policies focusing on mobility and heating to avoid a targeted number of pollution-related deaths: Forward and backward approaches combining air pollution modeling, health impact assessment and cost-benefit analysis. Environment International. 2022;159.

14. Buonocore JJ, Lambert KF, Burtraw D, Sekar S, Driscoll CT. An analysis of costs and health co-benefits for a U.S. Power Plant Carbon Standard. PLoS ONE. 2016;11(6):e0156308.

15. Burtraw D, Palmer K, Bharvirkar R, Paul A. Cost-effective reduction of NOx emissions from electricity generation. J Air Waste Manag Assoc. 2001;51(10):1476-89.

16. Cai W, Hui J, Wang C, Zheng Y, Zhang X, Zhang Q, et al. The Lancet Countdown on PM2.5 pollution-related health impacts of China's projected carbon dioxide mitigation in the electric power generation sector under the Paris Agreement: a modelling study. The Lancet Planetary Health. 2018;2(4):e151-e61.

17. Carnevale C, Ferrari F, Guariso G, Maffeis G, Turrini E, Volta M. Assessing the economic and environmental sustainability of a regional air quality plan. Sustainability (Switzerland). 2018;10(10).

18. Ćetković J, Lakić S, Žarković M, Đurović G, Vujadinović R. Application of economic analysis of air pollution reduction measures. Polish Journal of Environmental Studies. 2020;30(1):585-99.

19. Chau CK, Hui WK, Tse MS. Valuing the health benefits of improving indoor air quality in residences. Science of the Total Environment. 2008;394(1):25-38.

20. Chen F, Yamashita K, Kurokawa J, Klimont Z. Cost-benefit analysis of reducing premature mortality caused by exposure to ozone and PM2.5 in East Asia in 2020. Water, Air, and Soil Pollution. 2015;226(4).

21. Chen M. Whether it is economical to use combined heat and power (CHP) system for the efficient utilization of associated petroleum gas in oil extraction sites in China: A cost-benefit analysis considering environmental benefits. Frontiers in Environmental Science. 2022;10.

22. Chestnut LG, Mills DM, Cohan DS. Cost-benefit analysis in the selection of efficient multipollutant strategies. J Air Waste Manag Assoc. 2006;56(4):530-6.

23. Cropper ML, Guttikunda S, Jawahar P, Lazri Z, Malik K, Song X-P. Applying Benefit-Cost Analysis to Air Pollution Control in the Indian Power Sector. Journal of Benefit-Cost Analysis. 2019;10:185-205.

24. Evans JS, Rojas-Bracho L, Hammitt JK, Dockery DW. Mortality Benefits and Control Costs of Improving Air Quality in Mexico City: The Case of Heavy Duty Diesel Vehicles. Risk Analysis. 2021;41(4):661-77.

25. Feng T, Du H, Coffman DM, Qu A, Dong Z. Clean heating and heating poverty: A perspective based on cost-benefit analysis. Energy policy. 2021;152.

26. Fisk WJ, Chan WR. Effectiveness and cost of reducing particle-related mortality with particle filtration. Indoor Air. 2017;27(5):909-20.

27. Fisk WJ, Chan WR. Health benefits and costs of filtration interventions that reduce indoor exposure to PM2.5 during wildfires. Indoor Air. 2017;27(1):191-204.

28. Fung KM, Tai APK, Yong T, Liu X, Lam HM. Co-benefits of intercropping as a sustainable farming method for safeguarding both food security and air quality. Environmental Research Letters. 2019;14(4).

29. Giannakis E, Kushta J, Bruggeman A, Lelieveld J. Costs and benefits of agricultural ammonia emission abatement options for compliance with European air quality regulations. Environmental Sciences Europe. 2019;31(1).

30. Guo X, Zhao L, Chen D, Jia Y, Zhao N, Liu W, et al. Air quality improvement and health benefit of PM2.5 reduction from the coal cap policy in the Beijing–Tianjin–Hebei (BTH) region, China. Environmental Science and Pollution Research. 2018;25(32):32709-20.

31. Guo X, Jia C, Xiao B. Spatial variations of PM2.5 emissions and social welfare induced by clean heating transition: A gridded cost-benefit analysis. Science of the Total Environment. 2022;826.

32. Gupta A, Naved MM, Kumbhare H, Bherwani H, Das D, Labhsetwar N. Impact assessment of clean cookstove intervention in Gujarat, India: a potential case for corporate social responsibility (CSR) funding. Environmental Science and Pollution Research. 2021;28(10):12740-52.

33. Howard DB, Thé J, Soria R, Fann N, Schaeffer R, Saphores JDM. Health benefits and control costs of tightening particulate matter emissions standards for coal power plants - The case of Northeast Brazil. Environment International. 2019;124:420-30.

34. Hsieh IL, Chossière GP, Gençer E, Chen H, Barrett S, Green WH. An Integrated Assessment of Emissions, Air Quality, and Public Health Impacts of China's Transition to Electric Vehicles. Environ Sci Technol. 2022.

35. Huang J, Zhu Y, Kelly JT, Jang C, Wang S, Xing J, et al. Large-scale optimization of multi-pollutant control strategies in the Pearl River Delta region of China using a genetic algorithm in machine learning. Science of the Total Environment. 2020;722.

36. Hutchinson EJ, Pearson PJG. An evaluation of the environmental and health effects of vehicle exhaust catalysts in the United Kingdom. Environmental Health Perspectives. 2004;112(2):132-41.

37. Hutton G, Rehfuess E, Tediosi F. Evaluation of the costs and benefits of interventions to reduce indoor air pollution. Energy for Sustainable Development. 2007;11(4):34-43.

38. Irfan M, Cameron MP, Hassan G. Interventions to mitigate indoor air pollution: A cost-benefit analysis. PLoS ONE. 2021;16(9 September).

39. Isihak S, Akpan U, Adeleye M. Interventions for mitigating indoor-air pollution in Nigeria: A cost-benefit analysis. International Journal of Energy Sector Management. 2012;6(3):417-29.

40. Iwata K. Cost-Benefit Analysis of Enforcing Installation of Particulate Matter Elimination Devices on Diesel Trucks in Japan. Environmental Economics and Policy Studies. 2011;13(1):1-19.

41. Jeuland M, Tan Soo J-S, Shindell D. The Need for Policies to Reduce the Costs of Cleaner Cooking in Low Income Settings: Implications from Systematic Analysis of Costs and Benefits. Energy Policy. 2018;121:275-85.

42. Jin Y, Andersson H, Zhang S. China's Cap on Coal and the Efficiency of Local Interventions: A Benefit-Cost Analysis of Phasing Out Coal in Power Plants and in Households in Beijing. Journal of Benefit-Cost Analysis. 2017;8(2):147-86.

43. Kiely L, Spracklen DV, Arnold SR, Papargyropoulou E, Conibear L, Wiedinmyer C, et al. Assessing costs of Indonesian fires and the benefits of restoring peatland. Nature Communications. 2021;12(1):7044.

44. Kim SE, Xie Y, Dai H, Fujimori S, Hijioka Y, Honda Y, et al. Air quality co-benefits from climate mitigation for human health in South Korea. Environment International. 2020;136:105507.

45. Kiziltan A, Kiziltan M, Ara Aksoy S, Aydınalp Köksal M, Tekeli ŞE, Duran N, et al. Cost–benefit analysis of road-transport policy options to combat air pollution in Turkey. Environment, Development and Sustainability. 2022.

46. Krewitt W, Holland M, Trukenmüller A, Heck T, Friedrich R. Comparing costs and environmental benefits of strategies to combat acidification and ozone in Europe. Environmental Economics and Policy Studies. 1999;2(4):249-66.

47. Lai H-C, Hsiao M-C, Liou J-L, Lai L-W, Wu P-C, Fu JS. Using Costs and Health Benefits to Estimate the Priority of Air Pollution Control Action Plan: A Case Study in Taiwan. Applied Sciences-Basel. 2020;10(17).

48. Lange SS, Mulholland SE, Honeycutt ME. What Are the Net Benefits of Reducing the Ozone Standard to 65 ppb? An Alternative Analysis. Int J Environ Res Public Health. 2018;15(8).

49. Larson BA. The Economics of Air Pollution Health Risks in Russia: A Case Study of Volgograd. World Development. 1999;27(10):1803-19.

50. Lavee D. Cost-benefit analysis of implementing policy measures for reducing PM and O3 concentrations: the case of Israel. International Journal of Sustainable Development and World Ecology. 2018;25(8):682-94.

51. Levy JI, Biton L, Hopke PK, Zhang KM, Rector L. A cost-benefit analysis of a pellet boiler with electrostatic precipitator versus conventional biomass technology: A case study of an institutional boiler in Syracuse, New York. Environmental Research. 2017;156:312-9.

52. Li J, Guttikunda SK, Carmichael GR, Streets DG, Chang YS, Fung V. Quantifying the human health benefits of curbing air pollution in Shanghai. Journal of Environmental Management. 2004;70(1):49-62.

53. Li Y, Crawford-Brown DJ. Assessing the co-benefits of greenhouse gas reduction: Health benefits of particulate matter related inspection and maintenance programs in Bangkok, Thailand. Science of the Total Environment. 2011;409(10):1774-85.

54. Liu Y, Zhou B, Wang J, Zhao B. Health benefits and cost of using air purifiers to reduce exposure to ambient fine particulate pollution in China. Journal of Hazardous Materials. 2021;414.

55. Lomas J, Schmitt L, Jones S, McGeorge M, Bates E, Holland M, et al. A pharmacoeconomic approach to assessing the costs and benefits of air quality interventions that improve health: A case study. BMJ Open. 2016;6(6):e010686.

56. Lopez NS, Soliman J, Biona JBM, Fulton L. Cost-Benefit Analysis of Alternative Vehicles in the Philippines Using Immediate and Distant Future Scenarios. Transportation Research: Part D: Transport and Environment. 2020;82.

57. Lopez-Aparicio S, Grythe H, Thorne RJ, Vogt M. Costs and benefits of implementing an Environmental Speed Limit in a Nordic city. Science of the Total Environment. 2020;720.

58. Luo Q, Copeland B, Garcia-Menendez F, Johnson JX. Diverse Pathways for Power Sector Decarbonization in Texas Yield Health Cobenefits but Fail to Alleviate Air Pollution Exposure Inequities. Environmental Science and Technology. 2022;56(18):13274-83.

59. Malla MB, Bruce N, Bates E, Rehfuess E. Applying Global Cost-Benefit Analysis Methods to Indoor Air Pollution Mitigation Interventions in Nepal, Kenya and Sudan: Insights and Challenges. Energy Policy. 2011;39(12):7518-29.

60. Mao X, Guo X, Chang Y, Peng Y. Improving air quality in large cities by substituting natural gas for coal in China: Changing idea and incentive policy implications. Energy Policy. 2005;33(3):307-18.

61. Mardones C. Ex-post evaluation and cost-benefit analysis of a heater replacement program implemented in southern Chile. Energy. 2021;227.

62. Markandya A, Sampedro J, Smith SJ, Van Dingenen R, Pizarro-Irizar C, Arto I, et al. Health co-benefits from air pollution and mitigation costs of the Paris Agreement: a modelling study. The Lancet Planetary Health. 2018;2(3):e126-e33.

63. Mazorra J, Sanchez-Jacob E, de la Sota C, Fernandez L, Lumbreras J. A comprehensive analysis of cooking solutions co-benefits at household level: Healthy lives and well-being, gender and climate change. Science of the Total Environment. 2020;707.

64. Mesbah SM, Hakami A, Schott S. Optimal ozone reduction policy design using adjoint-based NOx marginal damage information. Environmental Science and Technology. 2013;47(23):13528-35.

65. Miraglia SG. Health, environmental, and economic costs from the use of a stabilized diesel/ethanol mixture in the city of São Paulo, Brazil. Cad Saude Publica. 2007;23:S559-69.

66. Miranda AI, Ferreira J, Silveira C, Relvas H, Duque L, Roebeling P, et al. A cost-efficiency and health benefit approach to improve urban air quality. Science of the Total Environment. 2016;569:342-51.

67. Moon H, Yoo SH, Huh SY. Monetary valuation of air quality improvement with the stated preference technique: A multi-pollutant perspective. Science of the Total Environment. 2021;793.

68. Netalieva I, Wesseler J, Heijman W. Health costs caused by oil extraction air emissions and the benefits from abatement: The case of Kazakhstan. Energy Policy. 2005;33(9):1169-77.

69. Nishioka Y, Levy JI, Norris GA, Bennett DH, Spengler JD. A risk-based approach to health impact assessment for input-output analysis. Part 2: Case study of insulation. International Journal of Life Cycle Assessment. 2005;10(4):255-62.

70. Nuhu P, Bukari D, Banye EZ. Driving improved cooking technology uptake in Ghana: An analysis of costs and benefits. Energy for Sustainable Development. 2022;66:26-43.

71. Okada A. Benefit, Cost, and Size of an Emission Control Area: A Simulation Approach for Spatial Relationships. Maritime Policy and Management. 2019;46(5):565-84.

72. Olsthoorn X, Amann M, Bartonova A, Clench-Aas J, Cofala J, Dorland K, et al. Cost benefit analysis of European air quality targets for sulphur dioxide, nitrogen dioxide and fine and suspended particulate matter in cities. Environmental and Resource Economics. 1999;14(3):333-51.

73. Ou Y, West JJ, Smith SJ, Nolte CG, Loughlin DH. Air pollution control strategies directly limiting national health damages in the US. Nature Communications. 2020;11(1).

74. Palmer K, Burtraw D, Shih JS. The benefits and costs of reducing emissions from the electricity sector. Journal of Environmental Management. 2007;83(1):115-30.

75. Pandey MD, Nathwani JS. Canada Wide Standard for particulate matter and ozone: cost-benefit analysis using a Life Quality Index. Risk Anal. 2003;23(1):55-67.

76. Perl LJ, Dunbar FC. Cost effectiveness and cost-benefit analysis of air quality regulations ( USA). American Economic Review. 1982;72(2):208-13.

77. Pisoni E, Volta M. Modeling Pareto efficient PM10 control policies in Northern Italy to reduce health effects. Atmospheric Environment. 2009;43(20):3243-8.

78. Raff Z, Walter JM. Evaluating the Efficacy of Ambient Air Quality Standards at Coal-Fired Power Plants. Journal of Agricultural and Resource Economics. 2020;45(3):428-44.

79. Rezazadeh AA, Alizadeh S, Avami A, Kianbakhsh A. Integrated analysis of energy-pollution-health nexus for sustainable energy planning. Journal of Cleaner Production. 2022;356.

80. Sampedro J, Smith SJ, Arto I, González-Eguino M, Markandya A, Mulvaney KM, et al. Health co-benefits and mitigation costs as per the Paris Agreement under different technological pathways for energy supply. Environment International. 2020;136.

81. Schucht S, Colette A, Rao S, Holland M, Schöpp W, Kolp P, et al. Moving towards ambitious climate policies: Monetised health benefits from improved air quality could offset mitigation costs in Europe. Environmental Science & Policy. 2015;50:252-69.

82. Shindell D, Ru M, Zhang Y, Seltzer K, Faluvegi G, Nazarenko L, et al. Temporal and spatial distribution of health, labor, and crop benefits of climate change mitigation in the United States. Proceedings of the National Academy of Sciences of the United States of America. 2021;118(46).

83. Stevens G, Wilson A, Hammitt JK. A benefit-cost analysis of retrofitting diesel vehicles with particulate filters in the Mexico City metropolitan area. Risk Analysis. 2005;25(4):883-99.

84. Suhyoung K, Chng LK. Cost–benefit analysis of pm2.5 policy in Korea. EnvironmentAsia. 2021;14(3):62-70.

85. Tang R, Zhao J, Liu Y, Huang X, Zhang Y, Zhou D, et al. Air quality and health co-benefits of China’s carbon dioxide emissions peaking before 2030. Nature Communications. 2022;13(1).

86. Thanh BD, Lefevre T. Assessing health benefits of controlling air pollution from power generation: the case of a lignite-fired power plant in Thailand. Environ Manage. 2001;27(2):303-17.

87. Tse MS, Chau CK, Lee WL. Assessing the benefit and cost for a voluntary indoor air quality certification scheme in Hong Kong. Science of the Total Environment. 2004;320(2):89-107.

88. Vandyck T, Keramidas K, Kitous A, Spadaro JV, Van Dingenen R, Holland M, et al. Air quality co-benefits for human health and agriculture counterbalance costs to meet Paris Agreement pledges. Nature Communications. 2018;9(1).

89. Voorhees AS, Araki S, Sakai R, Sato H. An ex post cost-benefit analysis of the nitrogen dioxide air pollution control program in tokyo. Journal of the Air and Waste Management Association. 2000;50(3):391-410.

90. Voorhees AS, Uchiyama I. Particulate matter air pollution control programs in Japan - An analysis of cost savings in the absence of future remediation. Journal of Risk Research. 2008;11(3):395-408.

91. Wagner S, Angenendt E, Beletskaya O, Zeddies J. Costs and benefits of ammonia and particulate matter abatement in German agriculture including interactions with greenhouse gas emissions. Agricultural Systems. 2015;141:58-68.

92. Wagner S, Angenendt E, Beletskaya O, Zeddies J. Assessing ammonia emission abatement measures in agriculture: Farmers' costs and society's benefits – A case study for Lower Saxony, Germany. Agricultural Systems. 2017;157:70-80.

93. West JJ, Fiore AM, Horowitz LW, Mauzerall DL. Global health benefits of mitigating ozone pollution with methane emission controls. Proceedings of the National Academy of Sciences of the United States of America. 2006;103(11):3988-93.

94. Wiser R, Millstein D. Evaluating the economic return to public wind energy research and development in the United States. Applied Energy. 2020;261.

95. Wu R, Dai H, Geng Y, Xie Y, Masui T, Liu Z, et al. Economic Impacts from PM2.5 Pollution-Related Health Effects: A Case Study in Shanghai. Environmental Science and Technology. 2017;51(9):5035-42.

96. Xie Y, Zhao L, Xue J, Hu Q, Xu X, Wang H. A cooperative reduction model for regional air pollution control in China that considers adverse health effects and pollutant reduction costs. Science of the Total Environment. 2016;573:458-69.

97. Yang W, Yu C, Yuan W, Wu X, Zhang W, Wang X. High-resolution vehicle emission inventory and emission control policy scenario analysis, a case in the Beijing-Tianjin-Hebei (BTH) region, China. Journal of Cleaner Production. 2018;203:530-9.

98. Zhang H, Zhang B, Bi J. More efforts, more benefits: Air pollutant control of coal-fired power plants in China. Energy. 2015;80:1-9.

99. Zhang J, Jiang H, Zhang W, Ma G, Wang Y, Lu Y, et al. Cost-benefit analysis of China’s Action Plan for Air Pollution Prevention and Control. Frontiers of Engineering Management. 2019;6(4):524-37.

100. Zhang S, An K, Li J, Weng Y, Zhang S, Wang S, et al. Incorporating health co-benefits into technology pathways to achieve China's 2060 carbon neutrality goal: a modelling study. The Lancet Planetary Health. 2021;5(11):e808-e17.

101. Zhao B, Zhao J, Zha H, Hu R, Liu Y, Liang C, et al. Health Benefits and Costs of Clean Heating Renovation: An Integrated Assessment in a Major Chinese City. Environmental Science and Technology. 2021;55(14):10046-55.

102. Zhao N, Elshareef H, Li B, Wang B, Jia Z, Zhou L, et al. The efforts of China to combat air pollution during the period of 2015-2018: A case study assessing the environmental, health and economic benefits in the Beijing-Tianjin-Hebei and surrounding "2 + 26" regions. Science of the Total Environment. 2022;853:158437.

103. Zhou J, Wang J, Jiang H, Cheng X, Lu Y, Zhang W, et al. Cost-benefit analysis of yellow-label vehicles scrappage subsidy policy: A case study of Beijing-Tianjin-Hebei region of China. Journal of Cleaner Production. 2019;232:94-103.

104. Zhou J, Jiang H, Cheng X, Lu Y, Zhang W, Dong Z. Are the Benefits of a High-Emission Vehicle Driving Area Restriction Policy Greater than the Costs? International Journal of Environmental Research and Public Health. 2022;19(23).
