# Supplementary material for: The costs, health and economic impact of air pollution control strategies: a systematic review
Source: Glob Health Res Policy. 2024 Aug 21;9:30. doi: 10.1186/s41256-024-00373-y (PMC11337783; doi:10.1186/s41256-024-00373-y)
Supplement: Supplementary file 5 — Additional file 5. [file 41256_2024_373_MOESM5_ESM.docx]

| Author |  |  |  |  |  |  |  |  |  |  |  |  |  |  |  |  |  |  |  |  |  |  |  |  |  |  |  |  |
| --- | --- | --- | --- | --- | --- | --- | --- | --- | --- | --- | --- | --- | --- | --- | --- | --- | --- | --- | --- | --- | --- | --- | --- | --- | --- | --- | --- | --- |
| Checklist | 1 | 2 | 3 | 4 | 5 | 6 | 7 | 8 | 9 | 10 | 11 | 12 | 13 | 14 | 15 | 16 | 17 | 18 | 19 | 20 | 21 | 22 | 23 | 24 | 25 | 26 | 27 | 28 |
| Aldred 2016 | X | X | X | X | - | X | X | - | X | - | X | X | X | X | X | X | - | X | - | X | - | X | - | X | - | X | X | X |
| Antturi 2016 | X | X | X | X | - | X | X | - | - | - | X | X | X | X | X | X | - | - | - | X | - | X | X | X | - | X | - | - |
| Åström 2018 | X | X | X | X | - | X | X | - | X | - | X | X | X | X | X | X | - | - | - | X | - | X | X | X | - | X | X | X |
| Aunan 1998 | X | X | X | X | - | X | X | - | X | - | X | X | X | X | X | X | - | - | - | - | - | X | X | - | - | X | - | - |
| Aunan 2013 | X | X | X | X | - | X | X | - | - | X | X | X | X | X | X | X | X | X | - | X | - | X | X | X | - | X | X | - |
| Babcock 1973 | X | X | X | - | - | X | X | - | X | - | X | X | X | X | - | - | - | - | - | X | - | X | X | X | - | X | X | - |
| Ballini 2015 | X | X | X | X | - | X | X | - | X | - | X | - | X | X | X | X | X | - | - | - | - | X | X | - | - | X | - | - |
| Barstow 2019 | X | X | X | X | - | X | X | - | X | - | X | X | X | X | - | X | X | - | - | X | - | X | X | X | - | X | X | X |
| Beatty 2011 | - | - | X | - | X | X | X | - | X | - | X | X | X | X | - | - | - | X | - | X | - | X | - | X | - | X | - | - |
| Bollen 2009 | X | X | X | X | - | X | X | - | X | X | X | X | X | X | - | X | - | - | - | X | - | X | - | X | - | X | - | - |
| Bonilla 2023 | - | X | X | - | - | X | X | - | - | X | X | X | X | - | - | - | - | - | - | X | - | - | X | X | - | X | X | - |
| Borjesson 2021 | X | - | X | X | - | X | X | - | X | - | X | X | X | X | X | - | - | - | - | - | - | - | X | - | - | X | - | X |
| Bouscasse 2022 | - | X | - | X | - | X | X | - | X | - | X | X | X | X | - | - | - | - | - | - | - | - | - | - | - | - | X | X |
| Buonocore 2016 | X | X | X | X | - | X | X | - | X | - | X | X | X | X | X | X | - | - | - | X | - | - | X | X | - | X | X | X |
| Burtraw 2001 | X | X | X | X | - | X | X | - | - | - | X | - | X | X | X | X | X | - | - | X | - | X | X | X | - | X | - | - |
| Cai 2018 | X | X | X | X | - | X | X | - | X | X | X | X | X | - | X | X | - | - | - | - | - | X | - | - | - | X | X | X |
| Carnevale 2018 | - | X | X | X | - | X | X | - | X | X | X | X | X | X | X | X | - | - | - | X | - | - | X | X | - | X | X | X |
| Ćetković 2020 | - | X | X | - | - | X | X | - | X | X | X | - | X | X | - | - | - | - | - | - | - | X | X | - | - | - | - | X |
| Chau 2008 | - | X | X | X | - | X | X | - | X | X | X | X | X | - | X | - | - | X | X | - | - | X | - | - | - | X | - | - |
| Chen 2015 | - | X | X | X | - | X | X | - | X | - | X | X | X | X | X | X | - | - | - | - | - | X | - | - | - | - | - | - |
| Chen 2022 | X | - | - | X | - | X | X | - | X | X | X | X | X | X | - | - | - | - | - | - | - | - | X | - | - | X | - | X |
| Chestnut 2006 | - | - | X | X | - | X | X | - | - | - | X | X | - | X | - | - | - | - | - | - | - | - | X | - | - | - | - | - |
| Cropper 2019 | - | X | X | X | - | X | X | - | X | X | X | X | X | X | X | X | - | - | - | X | - | X | X | X | - | - | - | - |
| Evans 2021 | - | X | X | X | - | X | X | - | X | X | X | X | X | X | X | - | - | - | - | - | - | X | X | - | - | X | - | - |
| Feng 2021 | X | X | X | X | - | X | X | - | X | - | X | X | X | X | - | X | - | - | - | - | - | - | X | - | - | - | X | X |
| Fisk 2017 | X | X | X | X | - | X | X | - | - | - | X | X | X | X | - | - | - | - | - | - | - | X | X | - | - | X | X | - |
| Fisk 2017 | X | X | X | X | - | X | X | - | X | - | X | X | X | X | X | X | - | - | - | - | - | X | X | - | - | X | X | - |
| Fung 2019 | - | X | X | X | - | X | X | - | - | X | X | X | X | X | X | X | X | - | - | - | - | X | X | - | - | - | X | - |
| Giannakis 2019 | X | - | X | X | - | X | X | - | X | - | X | X | X | X | X | X | - | - | - | - | - | X | X | - | - | X | X | X |
| Guo 2018 | X | - | X | X | - | X | X | - | X | - | X | X | X | X | X | X | - | - | - | - | - | - | X | - | - | - | X | - |
| Guo 2022 | X | X | X | X | - | X | X | - | - | - | X | X | - | - | X | X | - | X | - | X | - | - | X | X | - | X | X | X |
| Gupta 2021 | X | X | X | - | - | X | X | - | - | - | - | X | X | X | - | - | - | - | - | - | - | X | - | - | - | - | X | X |
| Howard 2019 | X | X | X | X | - | X | X | - | - | - | X | X | X | X | X | X | - | - | - | - | - | X | X | - | - | X | X | X |
| Hsieh 2022 | X | X | X | X | - | X | X | - | X | - | X | X | X | - | X | X | - | - | - | X | - | - | X | X | - | X | - | X |
| Huang 2020 | X | X | X | X | - | X | - | - | X | - | - | X | X | X | X | X | - | - | - | - | - | - | X | - | - | X | X | X |
| Hutchinson 2004 | X | X | - | X | - | X | X | - | X | X | X | X | X | X | X | - | - | - | - | - | - | X | X | - | - | - | - | - |
| Hutton 2007 | - | X | X | X | - | X | X | - | X | X | X | X | X | X | X | - | - | - | - | X | - | X | X | X | - | - | - | - |
| Irfan 2021 | - | - | X | X | - | X | X | - | X | X | - | X | X | X | X | - | - | - | - | X | - | X | X | X | - | X | X | X |
| Isihak 2012 | - | X | X | X | - | X | X | - | - | X | X | X | X | X | - | - | - | - | - | X | - | X | X | X | - | - | - | - |
| Iwata 2011 | X | X | X | - | - | X | X | - | X | X | - | X | - | X | - | X | - | - | - | X | - | - | X | X | - | - | X | - |
| Jeuland 2018 | X | X | X | X | - | X | X | - | - | X | X | X | X | X | - | X | - | - | - | - | - | X | X | - | - | - | - | - |
| Jin 2017 | X | X | X | X | - | X | X | - | X | X | X | X | X | X | X | X | - | - | - | X | - | - | X | X | - | X | - | - |
| Kiely 2021 | X | X | X | X | - | X | X | - | X | - | X | X | X | X | X | X | - | - | - | - | - | - | - | - | - | - | - | - |
| Kim 2020 | - | X | X | X | - | X | X | - | X | - | X | X | X | X | X | X | - | - | - | - | - | - | - | - | - | X | X | X |
| Kiziltan 2022 | X | X | X | X | - | X | X | - | X | X | X | X | X | X | X | - | - | - | - | - | - | X | X | - | - | X | X | X |
| Krewitt 1999 | - | X | X | X | - | X | X | - | X | X | X | X | X | X | X | X | - | - | - | X | - | X | X | X | - | - | X | - |
| Lai 2020 | - | - | X | X | - | X | X | - | X | - | X | X | X | X | - | X | - | - | - | - | - | - | X | - | - | - | X | X |
| Lange 2018 | - | X | X | X | - | X | - | - | - | - | - | - | X | X | X | - | - | - | - | - | - | - | X | - | - | - | X | X |
| Larson 1999 | - | - | X | X | - | X | X | - | - | - | - | X | X | - | X | - | - | - | - | - | - | X | X | - | - | - | - | - |
| Lavee 2018 | - | - | X | X | - | X | X | - | X | - | X | X | X | - | - | - | - | - | - | - | - | X | X | - | - | - | - | X |
| Levy 2017 | X | X | X | X | - | X | X | - | - | X | X | X | X | X | - | X | - | - | - | - | - | - | X | - | - | - | X | - |
| Li 2004 | - | X | X | X | - | X | X | - | X | - | X | X | X | X | X | X | - | - | - | - | - | X | X | - | - | - | - | - |
| Li 2011 | X | X | X | X | - | X | X | - | X | - | X | X | X | X | X | X | - | - | - | X | - | X | X | X | - | - | - | - |
| Liu 2021 | X | X | X | X | - | X | X | - | - | - | X | X | X | X | X | - | - | - | - | X | - | - | - | X | - | X | X | X |
| Lomas 2016 | - | X | X | X | - | X | X | - | X | X | X | X | X | - | X | - | - | - | - | - | - | X | X | - | - | X | X | X |
| Lopez 2020 a | X | X | X | X | - | X | X | - | - | - | - | X | X | X | - | X | - | - | - | - | - | - | - | - | - | - | X | - |
| Lopez 2020 b | X | X | X | X | - | X | X | - | - | - | X | X | X | X | X | X | - | - | - | X | - | - | X | X | - | - | - | X |
| Luo 2022 | X | X | X | X | - | X | X | - | - | X | - | X | X | - | X | X | - | X | X | - | - | - | - | - | - | - | X | X |
| Malla 2011 | - | X | X | X | - | X | X | - | X | X | X | X | X | X | - | - | - | - | - | - | - | X | X | - | - | - | X | - |
| Mao 2005 | - | - | X | X | - | X | X | - | - | - | - | X | X | X | X | - | - | - | - | X | - | X | X | X | - | - | - | - |
| Mardones 2021 | X | X | X | X | - | X | X | - | - | - | X | X | X | X | - | - | - | - | - | X | - | X | X | - | - | X | - | X |
| Markandya 2018 | X | X | X | X | - | X | X | - | X | X | X | X | X | X | - | X | - | - | - | X | - | - | X | X | - | X | X | X |
| Mazorra 2020 | X | - | - | X | - | X | X | - | X | - | X | X | X | X | - | - | - | - | - | - | - | - | X | - | - | X | X | X |
| Mesbah 2013 | - | X | X | - | - | X | X | - | - | - | - | X | X | X | - | X | - | - | - | - | - | - | X | - | - | - | - | X |
| Miraglia 2007 | - | X | X | X | - | X | X | - | - | - | X | X | X | X | X | - | - | - | - | - | - | - | X | - | - | - | X | - |
| Miranda 2016 | - | X | - | X | - | X | X | - | - | - | X | X | - | - | - | - | - | - | - | - | - | - | X | - | - | - | X | - |
| Moon 2021 | - | X | X | - | - | X | X | - | X | X | - | X | X | X | - | - | - | - | - | - | - | X | X | - | - | X | X | X |
| Netalieva 2005 | X | - | X | X | X | X | X | - | X | - | X | X | X | X | - | - | - | - | - | - | - | X | X | - | - | - | - | - |
| Nishioka 2005 | X | - | - | - | - | X | X | - | X | - | - | X | X | - | - | - | - | - | - | - | - | - | - | - | - | - | - | - |
| Nuhu 2022 | X | X | X | X | - | X | X | - | - | - | X | X | X | X | - | - | - | - | - | X | - | - | X | X | - | - | X | X |
| Okada 2019 | X | - | X | X | - | X | X | - | - | - | X | X | X | X | - | - | - | - | - | X | - | X | - | X | - | X | X | X |
| Olsthoorn 1999 | X | - | - | X | - | X | X | - | X | - | X | X | X | X | - | - | - | - | - | X | - | - | X | X | - | - | - | - |
| Ou 2020 | - | X | X | - | - | X | X | - | X | - | - | X | X | X | - | X | - | - | - | - | - | - | X | - | - | - | - | - |
| Palmer 2007 | - | X | X | X | - | X | X | - | X | - | - | X | - | X | X | - | - | - | - | X | - | X | X | X | - | X | - | - |
| Pandey 2003 | X | - | - | X | - | X | X | - | X | X | X | X | X | X | X | - | - | - | - | - | - | - | X | - | - | - | - | - |
| Perl 1982 | - | - | - | - | - | X | X | - | X | - | X | X | X | X | X | - | - | - | - | X | - | - | X | X | - | - | - | - |
| Pisoni 2009 | - | - | - | X | - | X | X | - | - | - | X | X | X | X | - | - | - | - | - | - | - | - | - | - | - | - | X | - |
| Raff 2020 | - | - | X | X | - | X | X | - | X | - | - | X | X | X | X | - | - | - | - | - | - | - | X | - | - | - | - | - |
| Rezazadeh 2022 | - | X | X | X | - | X | X | - | - | - | X | X | X | X | - | - | - | - | - | - | - | - | - | - | - | - | - | X |
| Sampedro 2020 | X | X | X | X | - | X | X | - | X | X | X | X | X | X | - | X | - | - | - | X | - | - | - | - | - | X | X | X |
| Schucht 2015 | - | X | X | X | - | X | X | - | X | - | X | X | X | - | - | - | - | - | - | - | - | X | X | - | - | X | X | - |
| Shindell 2021 | X | X | - | X | - | X | X | - | X | - | X | X | X | - | - | X | - | - | - | - | - | - | - | - | - | - | X | - |
| Stevens 2005 | X | - | X | X | - | X | X | - | X | X | X | X | X | X | X | - | - | - | - | X | - | X | X | X | - | - | - | - |
| Suhyoung 2021 | - | X | - | X | - | X | X | - | X | X | - | X | X | X | X | - | - | - | - | - | - | - | X | - | - | - | X | - |
| Tang 2022 | - | - | - | x | - | X | X | - | X | - | X | X | X | X | - | X | - | - | - | - | - | X | X | - | - | X | - | - |
| Thanh 2001 | X | X | - | X | - | X | X | - | X | - | X | X | X | - | X | - | - | - | - | X | - | X | X | X | - | - | - | - |
| Tse 2004 | X | - | - | X | - | X | X | - | - | - | X | X | X | X | X | - | - | - | - | X | - | X | X | X | - | - | X | - |
| Vandyck 2018 | X | - | - | X | - | X | X | - | X | X | X | X | X | - | X | X | - | - | - | X | - | - | - | - | - | - | - | - |
| Voorhees 2000 | X | X | - | X | - | X | X | - | X | - | X | X | X | X | X | - | - | - | - | X | - | X | X | X | - | X | - | - |
| Voorhees 2008 | X | - | - | X | - | X | X | - | - | - | - | X | X | X | X | - | - | - | - | - | - | X | X | - | - | - | - | - |
| Wagner 2015 | - | X | X | X | - | X | X | - | - | X | - | X | X | X | X | X | - | - | - | X | - | X | X | X | - | - | X | - |
| Wagner 2017 | - | X | X | X | - | X | X | - | - | X | X | X | X | X | X | X | - | - | - | X | - | X | X | X | - | - | X | - |
| West 2006 | X | - | - | X | - | X | X | - | X | X | X | X | X | - | - | X | - | - | - | - | - | - | X | - | - | X | X | X |
| Wiser 2020 | X | X | X | X | - | X | X | - | X | X | - | X | X | X | X | - | - | - | - | X | - | X | X | X | - | - | X | X |
| Wu 2017 | - | X | X | X | - | X | X | - | X | - | X | X | X | X | - | - | - | - | - | X | - | - | X | X | - | X | X | - |
| Xie 2016 | - | X | X | X | - | X | X | - | X | - | X | X | X | X | X | - | - | - | - | X | - | - | X | X | - | - | X | - |
| Yang 2018 | X | X | X | X | - | X | X | - | X | X | X | X | X | X | - | X | - | - | - | - | - | - | - | - | - | - | X | - |
| Zhang 2015 | - | - | X | X | - | X | X | - | X | - | X | X | X | X | X | - | - | - | - | X | - | - | X | X | - | X | X | - |
| Zhang 2019 | X | X | X | X | - | X | X | - | X | - | X | X | X | X | - | - | - | - | - | - | - | X | X | - | - | X | X | - |
| Zhang 2021 | - | X | X | X | - | X | X | - | X | X | X | X | X | X | - | X | - | - | - | - | - | - | - | - | - | X | X | X |
| Zhao 2021 | X | X | X | X | - | X | X | - | X | X | X | X | X | X | - | - | - | - | - | - | - | - | - | - | - | - | - | x |
| Zhao 2022 | - | - | X | X | - | X | X | - | X | - | X | X | X | X | - | - | - | - | - | - | - | - | X | - | - | - | X | x |
| Zhou 2019 | X | - | X | X | - | X | X | - | X | - | X | X | X | X | - | X | - | - | - | - | - | - | X | - | - | - | X | - |
| Zhou 2022 | X | - | X | X | - | X | X | - | X | X | X | X | X | X | - | - | - | - | - | - | - | - | X | - | - | X | X | X |
